# Functional disparities of malonyl-ACP decarboxylase between *Xanthomonas campestris* and *Xanthomonas oryzae*

**DOI:** 10.1128/aem.02436-24

**Published:** 2025-04-08

**Authors:** Mingfeng Yan, Yonghong Yu, Lizhen Luo, Jingtong Su, Jincheng Ma, Zhe Hu, Haihong Wang

**Affiliations:** 1 Institute of Plant Protection, Jiangxi Academy of Agricultural Sciences 205386, Nanchang, Jiangxi, China; 2 Guangdong Provincial Key Laboratory for Developmental Biology and Environmental Adaptation of Agricultural Organisms, College of Life Sciences, South China Agricultural University 98444, Guangzhou, Guangdong, China; 3 Guangdong Food and Drug Vocational College 587402, Guangzhou, Guangdong, China; University of Nebraska-Lincoln, Lincoln, Nebraska, USA

**Keywords:** *Xanthomonas campestris *pv. *campestris*, *Xanthomonas oryzae *pv. *oryzae*, fatty acid biosynthesis, malonyl-ACP decarboxylase, 3-ketoacyl-ACP synthases III

## Abstract

*Xanthomonas campestris* pv. *campestris* (*Xcc*) and *X. oryzae* pv. *oryzae* (*Xoo*) are crucial plant pathogenic bacteria, causing crucifer black rot and rice leaf blight, respectively. Both bacterial species encode a protein containing the YiiD_C domain, designated MadB, which exhibits an 87.5% sequence identity between their MadBs. The *madB* genes from either *Xoo* or *Xcc* successfully restored the growth defect in *Ralstonia solanacearum* and *Escherichia coli fabH* mutants *in vivo. In vitro* assays demonstrated that MadB proteins possess malonyl-ACP decarboxylase activity, although *Xcc* MadB exhibited lower activity compared with *Xoo* MadB. Mutation of *madB* in both *Xoo* and *Xcc* strains led to decreased pathogenicity in their respective host plants. Interestingly, the *Xoo madB* mutant exhibited a significant increase in branched-chain fatty acid production, whereas the *Xcc madB* mutant showed only minor changes in fatty acid composition. Despite the reduction in exopolysaccharide (EPS) synthesis due to *madB* mutation in both *Xoo* and *Xcc*, EPS production in the *Xoo madB* mutant could be restored by exogenous sodium acetate supplementation. In contrast, sodium acetate failed to restore EPS synthesis in the *Xcc madB* mutant. Biochemical and genetic analyses indicated that these divergent physiological roles arise from the distinct biochemical functions of MadB in the two bacteria. In *Xoo*, the fatty acid synthesis (FAS) pathway mediated by MadB operates independently of the FAS pathway mediated by FabH. Conversely, in *Xcc*, the FAS pathway mediated by FabH is the primary route, with MadB’s pathway serving a supplementary and regulatory role. Further analysis of gene organization and expression regulation of *madB* in both bacteria corroborates these distinctions.

**IMPORTANCE:**

Despite the high conservation of the mad gene within the Proteobacteria, the physiological roles of the Mad protein remain largely unclear. Xoo and Xcc are bacteria with very close phylogenetic relationships, both encoding malonyl-ACP decarboxylase (MadB). However, MadB demonstrates substantial physiological function variations between these two species. This study demonstrates that even in closely related bacteria, homologous genes have adopted different evolutionary pathways to adapt to diverse living environments, forming unique gene expression regulation mechanisms. This has led to the biochemical functional divergence of homologous proteins within their respective species, ultimately resulting in distinct physiological functions.

## INTRODUCTION

**Fig 1:**
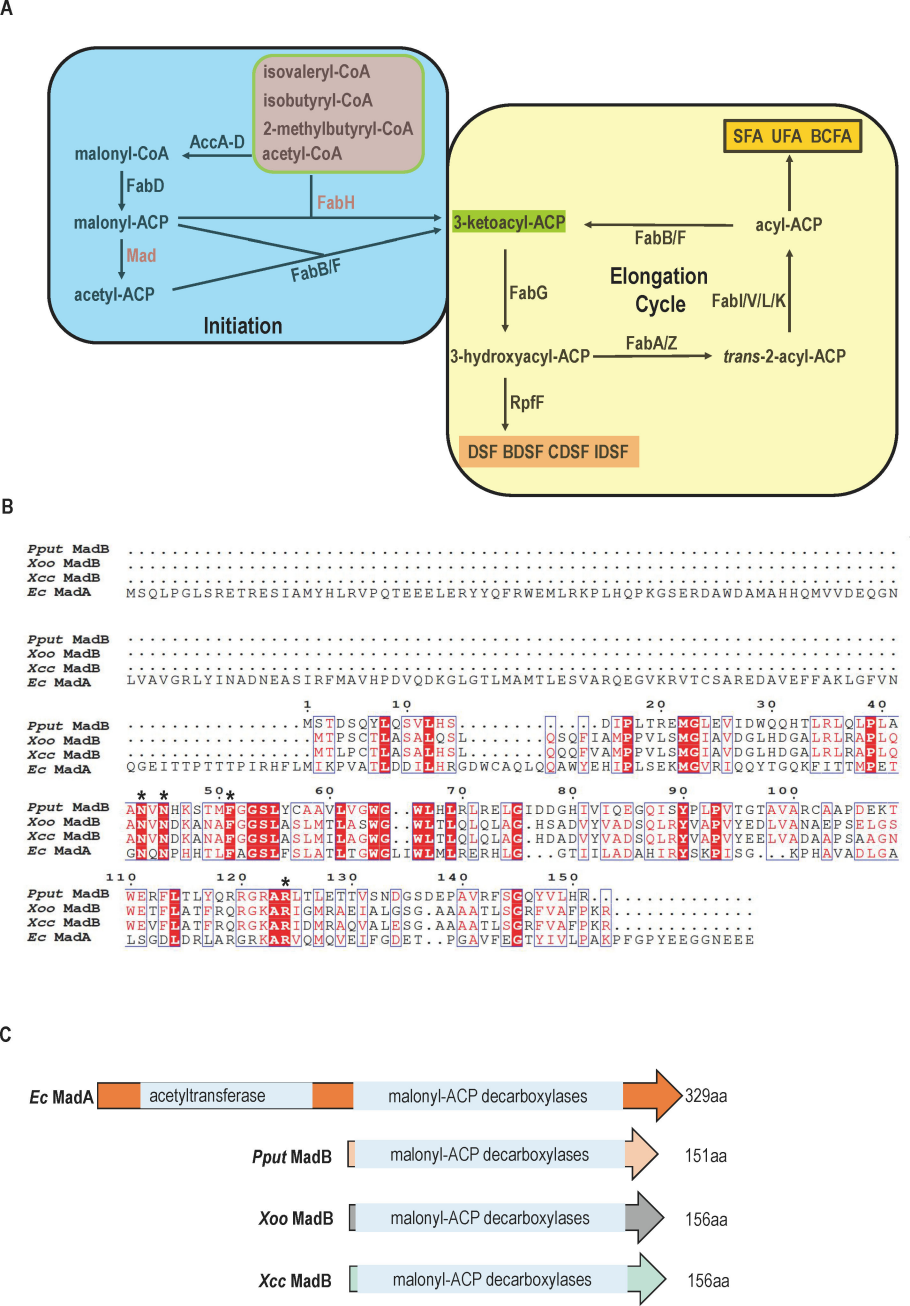
Fatty acid biosynthetic pathway in bacteria and MadB protein in *Xoo* and *Xcc*. (**A**) Fatty acid biosynthetic pathway in bacteria. Abbreviations: AccA-D, acetyl-CoA carboxylase; FabD, malonyl- CoA:ACP transacylase; Mad, malonyl-ACP decarboxylase (MadB from *Pseudomonas putida*, MadA from *Escherichia coli*); FabB, 3-ketoacyl-ACP synthase I; FabF, 3-ketoacyl-ACP synthase II; FabH, 3-ketoacyl ACP synthase III; FabG, 3-ketoacyl-ACP reductase; FabA/Z, 3-hydroxydecanoyl-ACP dehydratase/isomerase; FabI/V/L/K, enoyl-ACP reductase; RpfF, DSF synthase; SFA, straight-chain fatty acid; UFA, unsaturated fatty acid; BCFA, branch-chain fatty acid; DSF, BDSF, CDSF and IDSF, DSF-family signals. (**B**) Sequence alignments of Mad proteins. The alignments were performed with ESPript (https://espript.ibcp.fr/ESPript/ESPript/). White characters with red backgrounds are 100% identical, and red characters with blue frames are similar. Asterisks indicate conserved amino acid residues in Mad proteins. Abbreviations: *Xoo*, *X. oryzae* pv. *oryzae; Xcc*, *X. campestris* pv. *campestris; Pput*, *P. putida; Ec*, *E. coli*. (**C**) Carboxy-terminal decarboxylase domain predicted in *Xoo* MadB and *Xcc* MadB proteins.

Fatty acid synthesis (FAS) is a vital metabolic pathway that serves a central function in both eukaryotes and bacteria (1). Most bacteria utilize the Type II fatty acid synthase system (FAS II) for the synthesis of fatty acids, a process that depends on a series of distinct, small, soluble proteins (2–4) (Fig. 1A). Within FAS II, each enzyme, encoded by an individual gene, catalyzes a single step in the biosynthetic process (3, 4). This modular system enables the production of essential cellular components, such as membrane phospholipids, lipoglycans, and lipoproteins (4, 5). Additionally, it allows for the diversion of intermediates to synthesize other essential end-products, including lipid A (5, 6), quorum-sensing signal molecules (5, 7, 8), and vitamin cofactors (5, 9, 10). Consequently, delving into the fatty acid biosynthetic pathway of pathogenic bacteria could aid in identifying novel targets for antimicrobial drug development (2, 3).

The bacterial FAS process generally involves two stages: initiation and elongation (4, 5) (Fig. 1A). During the initiation phase, the typical 3-ketoacyl-ACP synthase (KASIII) catalyzes the condensation of acetyl-CoA with malonyl-ACP, resulting in the formation of the initial 3-ketoacyl-ACP intermediate, acetoacetyl-ACP (4, 5). *E. coli* FabH, the first identified KASIII, initiates the straight-chain FAS (11). Subsequently, in certain gram-positive bacteria like *Bacillus subtilis*, FabH-type KASIII has been found to catalyze the initiation of branch-chain FAS through the condensation of branch-chain acyl-CoA (including isobutyryl-CoA, isovaleryl-CoA, and 2-methylbutyryl-CoA) with malonyl-ACP (12). FabH is crucial for bacterial growth and survival; its absence leads to defective growth and changes in the fatty acid profile in *E. coli* (13), and a 90% reduction in FAS capacity in *Lactococcus lactis* (11, 14). Therefore, it was widely believed that most bacteria relied on FabH-type KASIII for FAS initiation until the discovery of malonyl-ACP decarboxylase (Mad)-mediated initiation in an *E. coli fabH* mutant (15, 16).

The newly discovered FAS initiation mechanism involves malonyl-ACP decarboxylase (Mad) catalyzing the decarboxylation of malonyl-ACP to yield acetyl-ACP (15, 17) (Fig. 1A). Subsequently, long-chain 3-ketoacyl-ACP synthase (KASI/II) facilitates the condensation of acetyl-ACP with malonyl-ACP, producing the initial 3-ketoacyl-ACP intermediate, acetoacetyl-ACP (15, 17). Given that functional homologs of Mad are prevalent across numerous bacterial species, this widely distributed alternative fatty acid initiation pathway offers new opportunities for targeting diverse biotechnological applications (17).

The *Xanthomonas* genus represents one of the most widespread and devastating groups of plant-associated bacterial pathogens, infecting at least 124 monocotyledonous and 268 dicotyledonous plant species (18, 19). A significant number of these affected species are economically crucial plants (19). Among the notable members of this genus are *X. campestris* pv. *campestris* (*Xcc*) and *X. oryzae* pv. *oryzae* (*Xoo*). *Xcc* is the causative agent of black rot disease, a highly destructive affliction affecting cruciferous vegetables globally (19, 20). In contrast, *Xoo* is the primary pathogen responsible for bacterial blight in rice, causing significant damage, particularly in tropical regions (20, 21). Consequently, understanding the biology and pathogenesis of these pathogens is crucial for developing effective disease management strategies, thereby safeguarding crop yields and ensuring agricultural productivity (18, 19).

During infections, both *Xoo* and *Xcc* produce a variety of virulence-related factors, including extracellular enzymes, exopolysaccharide (EPS), iron-chelating siderophores, membrane-bound yellow pigment xanthomonadin, and the type III secretion system (T3SS) with its effectors (19). However, the production of these virulence-related factors is regulated by quorum sensing (QS), which is mediated by diffusible signal factor (DSF) family signals (18, 22). The *rpf* gene cluster (*rpfBFCG*), which encodes fatty acyl-CoA ligase (RpfB), DSF synthase (RpfF), sensor (RpfC), and response regulator (RpfG), plays a crucial role in the quorum-sensing regulation of *Xcc* and *Xoo* (18, 22, 23). Four distinct DSF-family signals have been identified in *Xcc* and *Xoo*: DSF (*cis*-11-methyl-2-dodecenoic acid), BDSF (*cis*-2-dodecenoic acid), IDSF (*cis*-10-methyl-2-dodecenoic acid), and CDSF (*cis*, *cis*-11-methyldodeca-2, 5-dienoic acid) (24–26) (Fig. 1A). Research on 3-ketoacyl-ACP synthetase III (FabH) has shown that the bacterial FAS pathway in *Xcc* and *Xoo* provides precursors necessary for producing DSF-family signals (27, 28) (Fig. 1A). However, although both *Xcc* and *Xoo* FabH are involved in branched-chain FAS (Fig. 1A), the phenotypes of *fabH* mutations differ between these two species (27, 28). In *Xcc*, *fabH* is an essential gene, and its deletion results in lethal consequences (28). In contrast, an *Xoo fabH* deletion mutant remains viable and continues to synthesize straight-chain fatty acids (27). This suggests the potential existence of a novel FAS initiation pathway in *Xoo*.

In this paper, we identified YiiD domain-encoded proteins in both *Xcc* and *Xoo* and found that these proteins possess the ability to catalyze malonyl-ACP decarboxylation, which facilitates the initiation of fatty acid synthesis. However, the physiological functions of these proteins exhibit distinct characteristics in *Xoo* and *Xcc*.

## RESULTS

### Both *Xoo* and *Xcc* encode a putative protein containing the YiiD_C domain

Previous research has shown that the gene PXO_02706 in the *Xoo* PXO99A genome encodes 3-ketoacyl-ACP synthase III (KASIII, also known as FabH1), a key enzyme in the biosynthesis of branched-chain fatty acids in *Xoo* (27). This finding is consistent with studies on FabH in *Xcc* (28). Notably, *fabH* was identified as an essential gene in *Xcc*, and its deletion led to a complete loss of viability (28). Conversely, the Δ*fabH1* mutant in *Xoo* exhibited no significant growth impairment (27), suggesting that *Xoo* can survive without the KASIII enzyme. These observations indicate that *Xcc* predominantly relies on the FabH-mediated pathway for the initiation of fatty acid synthesis (FAS) (28), whereas *Xoo* likely employs an alternative unique pathway in addition to the FabH pathway for the same purpose.

Given that *fabH* is non-essential in *E. coli* and that Mad proteins can mediate FabH-independent initiation of FAS in both *E. coli* and *Pseudomonas putida* (16, 17, 29) (Fig. 1A), we investigated whether *Xoo* utilizes a Mad homolog for this process. A BLAST analysis using *E. coli* MadA and *P. putida* MadB as query sequences identified a putative homolog, PXO_03973 (designated *Xoo* MadB) (Fig. 1B). This protein was previously annotated as a hypothetical protein containing a YiiD_C domain (Fig. 1B and C). Protein sequence alignments revealed that *Xoo* MadB only shares 29.2% identity with the decarboxylase domain of *E. coli* MadA (15) and 34% identity with *P. putida* MadB, which contains a standalone decarboxylase domain necessary for malonyl-ACP decarboxylase activity (17) (Fig. 1B and C). Despite the low sequence identity, *Xoo* MadB retains highly conserved amino acid residues, including Asn 43, Asn 45, Phe 51, and Arg 124 (numbered according to the amino acid sequence of *P. putida* MadB [17]) (Fig. 1B), which are crucial for Mad protein function. These findings suggest that *Xoo* MadB may mediate a FabH-independent pathway for the initiation of FAS.

Interestingly, when *Xoo* MadB was used as a query sequence, BLAST analysis identified that XC_0210 encodes a hypothetical protein containing a YiiD_C domain in the *Xcc* 8004 genome, sharing 87.5% identity with *Xoo* MadB (Fig. 1B and C). This intriguing discovery raises questions about whether both *Xcc* and *Xoo* MadB catalyze the decarboxylation of malonyl-ACP and participate in the initiation of FAS *in vivo*, and why *fabH* is essential for *Xcc*. Further investigation is needed to elucidate the roles and mechanisms of these proteins in the respective organisms.

### Both *Xoo madB* and *Xcc madB* encode active malonyl-ACP decarboxylase

**Fig 2:**
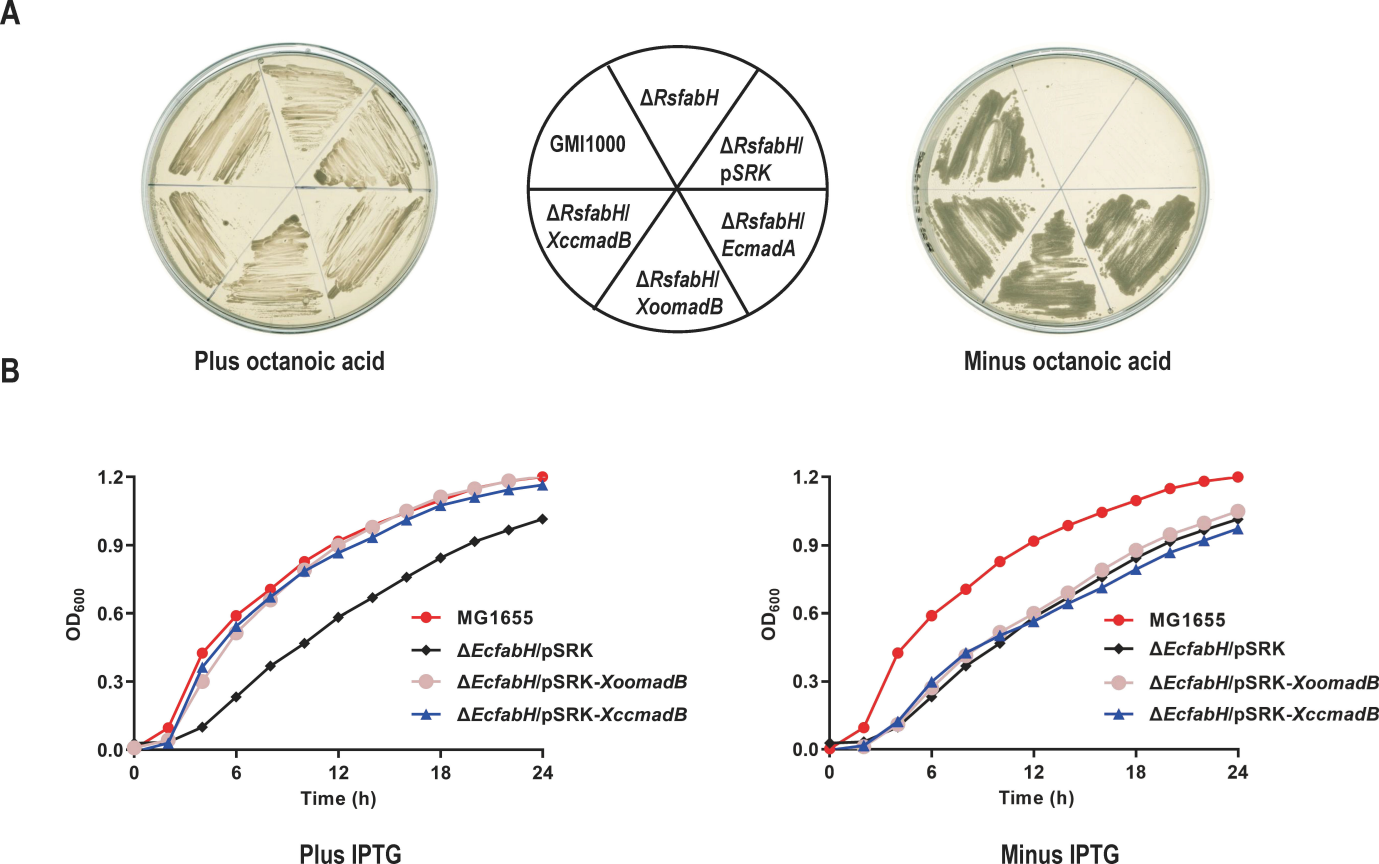
Complementation of *R. solanacearum* and *E. coli* mutant strains with *Xoo madB* or *Xcc madB*. (**A**) Growth of *R. solanacearum* mutant strains on BG plate. GMI1000, *R. solanacearum* wild-type strain; Δ*RsfabH*, *R. solanacearum fabH* deletion mutant; Δ*RsfabH*/pSRK, Δ*RsfabH* carrying empty vector pSRK-Tc; Δ*RsfabH*/*EcmadA*, Δ*RsfabH* carrying pSRK-Tc encoding *E. coli madA*; Δ*RsfabH*/*XoomadB*, Δ*RsfabH* carrying pSRK-Tc encoding *Xoo madB*; Δ*RsfabH*/*EcmadA*, Δ*RsfabH* carrying pSRK-Tc encoding *XccmadB*. (**B**) Growth of *E. coli fabH* mutant strains in LB liquid. MG1655, *E. coli* wild-type strain; Δ*EcfabH*/pSRK, Δ*EcfabH* carrying empty vector pSRK-Tc; Δ*EcfabH*/pSRK-*XoomadB*, Δ*EcfabH* carrying pSRK-Tc encoding *Xoo madB*; Δ*EcfabH*/pSRK-*XccmadB*, Δ*EcfabH* carrying pSRK-Tc encoding *Xcc madB*.

To identify and validate the *Xoo madB* and *Xcc madB* genes as encoding malonyl-ACP decarboxylase, DNA fragments of *Xoo madB* and *Xcc madB* were individually inserted into the high-copy number isopropyl-β-D-thiogalactoside (IPTG)-inducible vector pSRK-Tc (30). The resulting recombinant plasmids were then introduced into the *Ralstonia solanacearum fabH* deletion mutant (Δ*RsfabH*) to evaluate the role of the *Xoo* MadB and *Xcc* MadB proteins in the initiation of FAS. The *R. solanacearum* Δ*RsfabH* strain, which has the *fabH* gene in-frame deleted and consequently lost the ability to initiate FAS using acetyl-CoA as a precursor, is auxotrophic for octanoic acid (31) (Fig. 2A). The results showed that like *E. coli madA*, both *Xoo madB* and *Xcc madB* restored the growth of the Δ*RsfabH* strain on BG medium in the absence of octanoic acid supplementation. In contrast, Δ*RsfabH* carrying only the vector failed to grow under the same conditions (Fig. 2A). Additionally, when Asn45 (numbered according to the amino acid sequence of *P. putida* MadB), a key residue essential for the decarboxylase activity of Mad proteins (17), was substituted with Ala in *Xoo* MadB and *Xcc* MadB, the mutated *madB* genes lost their ability to complement the *fabH* mutant strains of *R. solanacearum* (Fig. S1).

The *E. coli fabH* mutant (Δ*EcfabH*) exhibited slow-growth phenotypes due to the absence of the major initiation enzyme FabH (13, 16) (Fig. 2B). Subsequent heterologous expression of *Xoo madB* and *Xcc madB* from the pSRK-Tc vector in the Δ*EcfabH* mutant showed that both genes restored Δ*EcfabH* mutant growth to wild-type levels in LB medium under IPTG induction (Fig. 2B). Conversely, neither *Xoo madB* nor *Xcc madB* could restore the growth of Δ*EcfabH* mutant without IPTG induction (Fig. 2B). These results indicated that both *Xoo madB* and *Xcc madB* could functionally replace *R. solanacearum* or *E. coli fabH* and played roles in the initiation of FAS, suggesting that both *Xoo madB* and *Xcc madB* likely encode functional malonyl-ACP decarboxylase.

The *E. coli* strain Δ*fabH*Δ*madA*/pRP-EcmadA, which harbors the temperature-sensitive plasmid pRP-EcmadA, is a temperature-sensitive mutant that can eliminate the plasmid pRP-EcmadA and grow at 42°C if the introduced gene in *trans* can functionally substitute for the *E. coli fabH* or *madA* genes. To further substantiate the role of *Xoo* MadB and *Xcc* MadB in the initiation of FAS, an IPTG-inducible vector carrying *Xoo madB* or *Xcc madB* was introduced into *E. coli* ∆*fabH*∆*madA*/pRP-EcmadA to screen for strains that lose pRP-EcmadA at 42°C. In the presence of IPTG, the ∆*fabH*∆*madA*/pSRK-XoomadB and ∆*fabH*∆*madA*/pSRK-XccmadB strains were screened (Fig. S2). These observations indicate that the overexpression of *Xoo madB* or *Xcc madB* rescued the synthetic lethality of *fabH* and *madA* in *E. coli* ∆*fabH*∆*madA*, thereby strongly suggesting that both *Xoo madB* and *Xcc madB* encode malonyl-ACP decarboxylase.

### Both *Xoo* MadB and *Xcc* MadB exhibit malonyl-ACP decarboxylase activity *in vitro*

To assess the malonyl-ACP decarboxylase activity of *Xoo* MadB or *Xcc* MadB *in vitro*, we expressed both proteins in *E. coli* BL21(DE3), and the C-terminally His_6_-tagged versions of the MadB proteins were successfully purified using nickel chelate chromatography. Additionally, we purified fatty acid biosynthetic proteins from *E. coli* (including FabH, FabB, FabG, FabA, FabI, *apo*-ACP, and *holo*-ACP) as well as *B. subtilis* Sfp (surfactin phosphopantetheinyl transferase) and *Vibrio harveyi* AasS (acyl-ACP synthetase) using the same methods (32).

**Fig 3:**
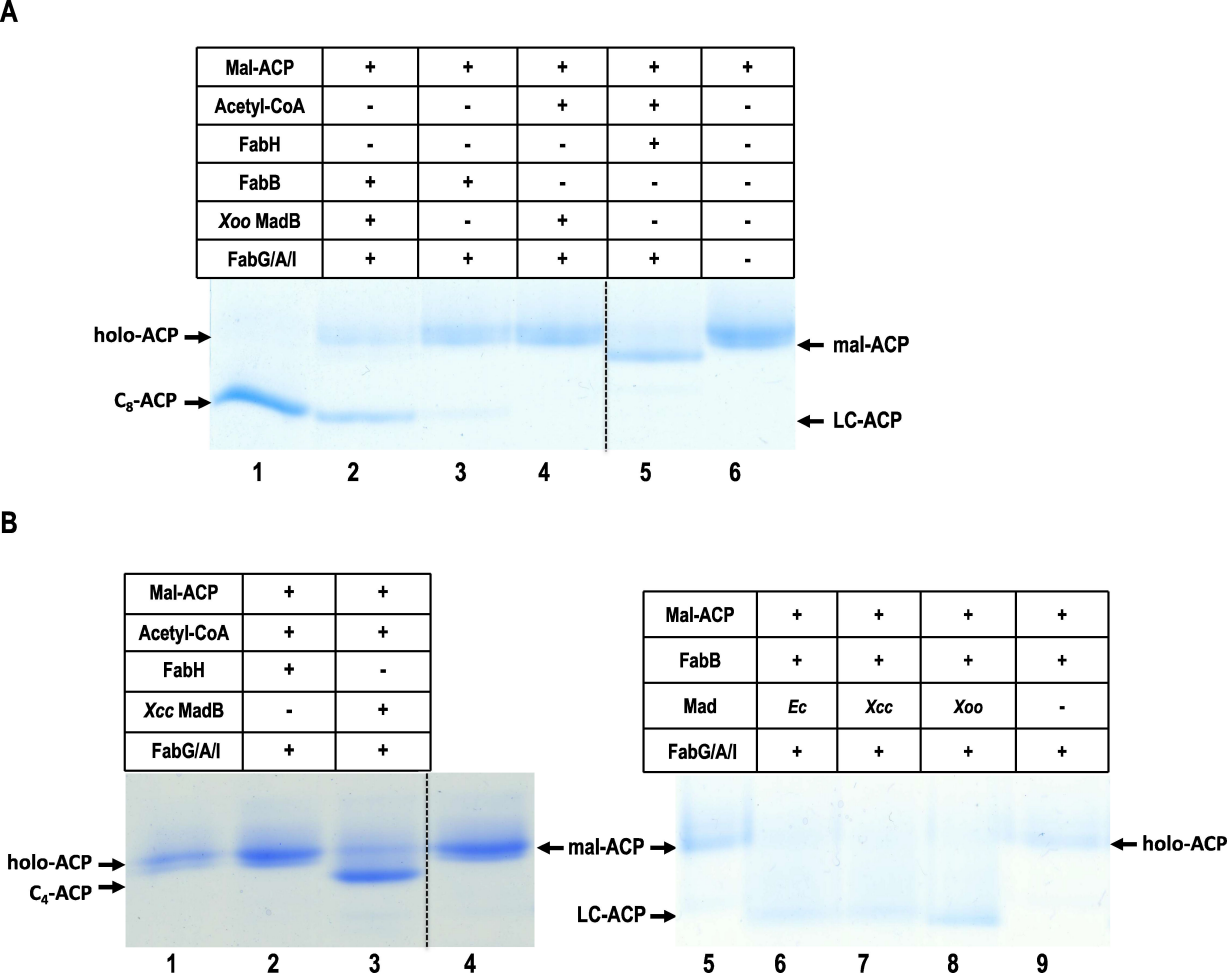
Enzymatic characterization of *Xoo* MadB and *Xcc* MadB in fatty acid biosynthesis *in vitro*. (**A**) Fatty acid synthesis was reconstructed to test malonyl-ACP decarboxylase activity of *Xoo* MadB. (**B**) Fatty acid synthesis was reconstructed to test malonyl-ACP decarboxylase activity of *Xcc* MadB. mal-ACP, malonyl-ACP; C_4:0_-ACP butyryl-ACP; C_8:0_-ACP, octanoyl-ACP; LC-ACP, long-chain acyl-ACP.

First, we examined the ability of the MadB proteins to catalyze the condensation of acetyl-CoA with malonyl-ACP. To reconstitute the initiation of FAS, we added MadB (or FabH), FabG, FabA, and FabI to a reaction mixture containing acetyl-CoA, malonyl-ACP, and NADPH, NADH, and analyzed the products using conformationally sensitive gel electrophoresis. The results showed that the reaction mixture containing FabH successfully produced butyryl-ACP (Fig. 3A, lane 5 and Fig. 3B, lane 2). However, reaction mixtures containing either *Xoo* MadB or *Xcc* MadB did not generate butyryl-ACP (Fig. 3A, lane 4 and Fig. 3B, lane 3), indicating that both proteins were unable to catalyze the condensation of acetyl-CoA with malonyl-ACP.

Next, we assessed the malonyl-ACP decarboxylase activity of MadB. Distinguishing acetyl-ACP from malonyl-ACP on the conformationally sensitive gel was challenging (33, 34), preventing us from directly detecting acetyl-ACP production to evaluate the decarboxylation activity of MadB. Since *E. coli* FabB can condense acetyl-ACP with malonyl-ACP to produce long-chain acyl-ACP *in vitro* (35, 36), we used malonyl-ACP as the substrate and reconstituted the FAS reaction by adding either *Xoo* MadB or *Xcc* MadB, along with FabB, FabG, FabA, and FabI. The reaction mixture containing MadB produced long-chain acyl-ACPs (Fig. 3A, lane 2 and Fig. 3B, lane 7), whereas the control reaction without MadB failed to generate acyl-ACPs (Fig. 3A, lanes 3 and Fig. 3B, lanes 9), indicating that both *Xoo* MadB and *Xcc* MadB exhibit malonyl-ACP decarboxylase activity.

Finally, we assayed the decarboxylase activities of *Xoo* MadB and *Xcc* MadB by monitoring the reduction in NADPH absorbance at 340 nm within a reaction mixture containing malonyl-ACP, NADPH, FabB, FabG, and MadB. Although both *Xoo* MadB and *Xcc* MadB exhibited malonyl-ACP decarboxylase activity, the specific activity of *Xcc* MadB (86.99 ± 9.19 nmol/µg per s) was only 65% of that of *Xoo* MadB (135.41 ± 12.51 nmol/µg per s). This suggests that the malonyl-ACP decarboxylase activity of *Xcc* MadB is significantly lower than that of *Xoo* MadB.

### *madB* is essential for the pathogenicity of both *Xoo* and *Xcc* in host plants

**Fig 4:**
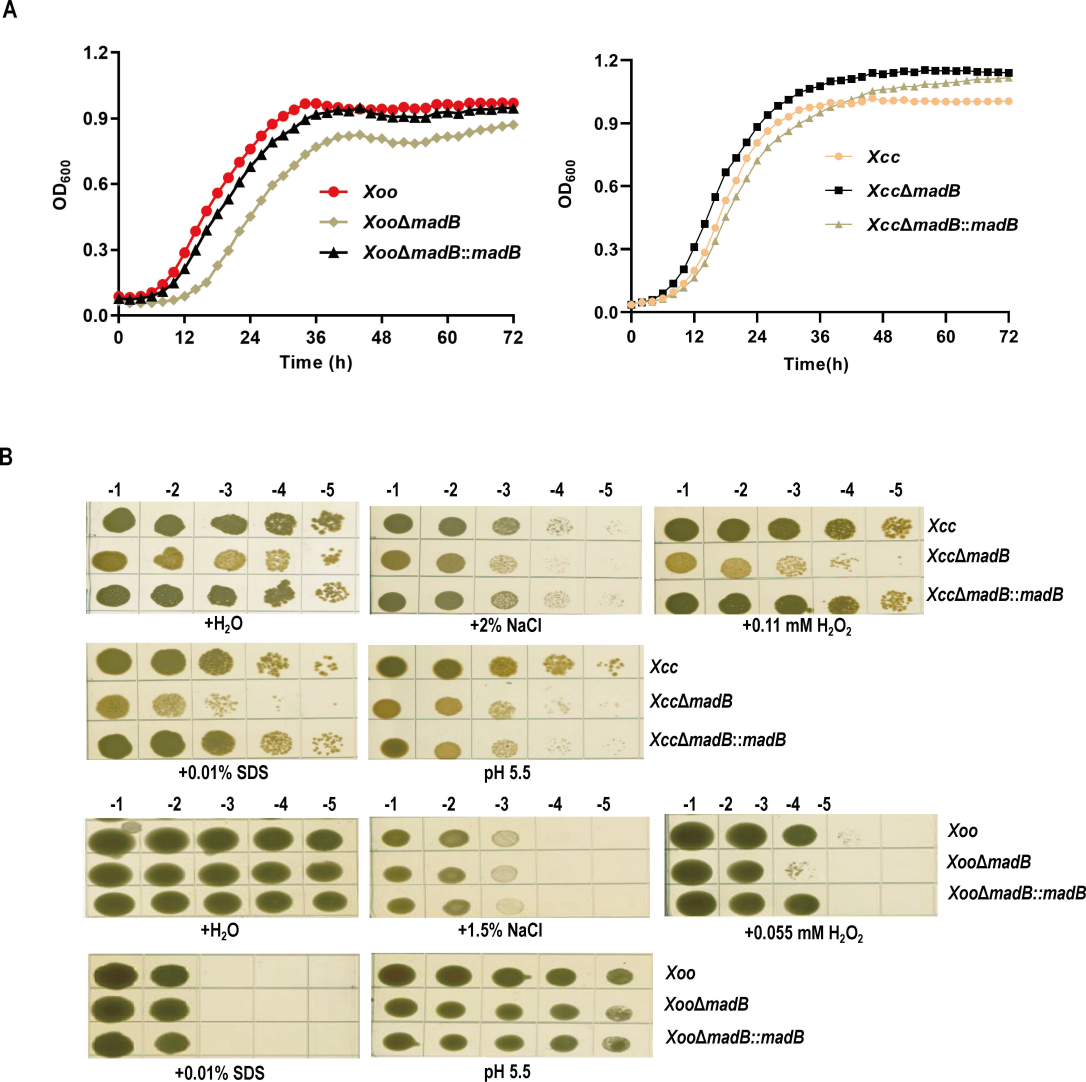
Growth responses of *madB* mutants under environmental stressors. (**A**) Growth of *madB* mutants in liquid medium. *Xoo* strains grew in NA medium; *Xcc* strains grew in nutrient glycerol (NYG) medium. (**B**) The growth of *madB* mutant strains under multiple stress factors. Stress factors include H_2_O_2_, sodium dodecyl sulfate (SDS), low pH, and high salt concentration.

To further elucidate the functions of MadB, mutant strains with deletions in the *Xoo madB* or *Xcc madB* gene were generated through allelic replacement (37). Both the *Xoo* Δ*madB* and *Xcc* Δ*madB* mutants remained viable, indicating that *madB* is not essential for the survival of either *Xoo* or *Xcc*. However, growth rates of the mutant strains exhibit slight variations. The *Xoo* Δ*madB* mutant grew slightly slower compared with the wild-type *Xoo* PXO99A strain (Fig. 4A, left), whereas the growth rate of the *Xcc* Δ*madB* mutant was nearly indistinguishable from that of the wild-type *Xcc* 8004 strain (Fig. 4A, right). Subsequently, the growth responses of *Xoo* Δ*madB* and *Xcc* Δ*madB* to various environmental stressors, including H_2_O_2_, sodium dodecyl sulfate (SDS), low pH, and high salt concentration, were evaluated. The results showed that the *Xcc* Δ*madB* mutant was more sensitive to NaCl, H_2_O_2_, SDS, and pH treatments than the wild-type *Xcc* 8004, whereas the *Xoo* Δ*madB* mutant showed increased sensitivity only to H_2_O_2_ (Fig. 4B). To understand the mechanism behind these changes, the membrane permeability of *madB* mutants was examined by measuring the uptake of the fluorescent dye 1-N-phenylnaphthylamine (NPN). The uptake kinetics of NPN showed that the deletion of *madB* did not alter the membrane permeability of *Xoo* but significantly increased the membrane permeability of the *Xcc madB* mutant (Fig. S3). This suggests that *madB* affects cell growth and response to environmental stress, likely by modulating membrane permeability.

**Fig 5:**
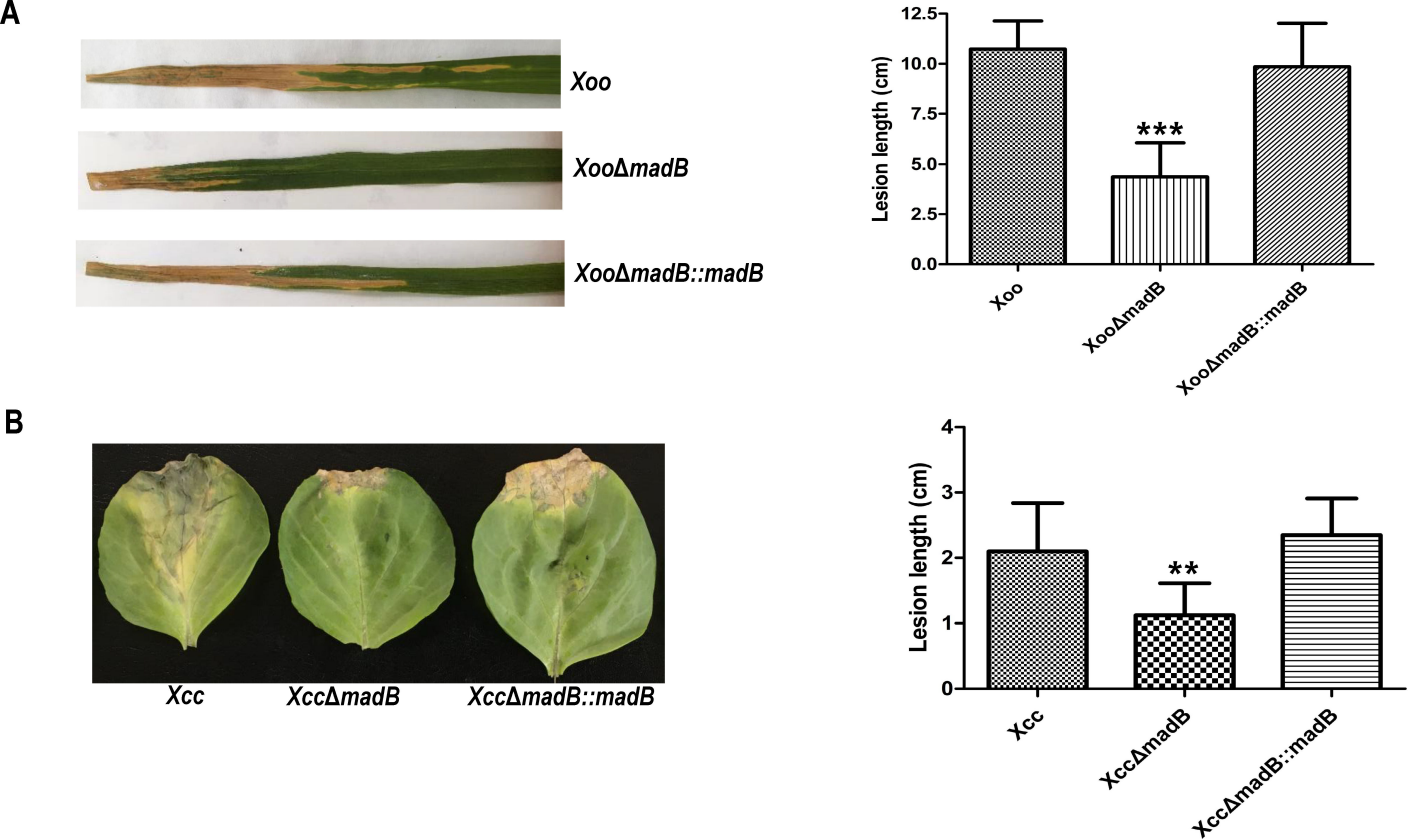
Inactivation of *madB* caused a deficiency in the virulence of *Xoo* or *Xcc*. (**A**) Virulence test of *Xoo madB* mutant strains on the host plant. Nipponbare (*Oryza sativa* L. *japonica* cv.) was used as a susceptible plant, and disease symptoms were recorded 14 dpi. (**B**) Virulence test of *Xcc madB* mutant strains on the host plant. *Brassica oleracea* “Jingfeng No. 1” was employed as the susceptible host, and disease symptoms were recorded 14 dpi. Statistical analyses were performed in GraphPad Prism 7 with multiple comparisons performed using ordinary one-way analysis of variance (ANOVA). The values are presented as the mean ± SD from three independent experiments. The asterisks above the error bars indicate significant differences compared with the wild-type strain (^**^P < 0.01, ^***^P < 0.001). All experiments were repeated three times with similar results.

To assess the contribution of *madB* to the virulence of both *Xcc* and *Xoo*, a leaf-clipping virulence assay was conducted. For the *Xoo* virulence test, Nipponbare (*Oryza sativa* L. *japonica* cv.) was used as a susceptible plant, and disease symptoms were recorded 14 days post-inoculation (dpi) (38). The wild-type PXO99A strain induced an average lesion length of 10.7 ± 1.4 cm at 14 dpi. In contrast, the *Xoo* Δ*madB* mutant produced significantly reduced lesion lengths (4.6 ± 1.6 cm) across all infected leaves, whereas the complementary strain *Xoo* Δ*madB::madB* exhibited symptoms comparable with those of the wild-type strain (Fig. 5A). For the *Xcc* virulence test, cabbage plants (*Brassica oleracea* ‘Jingfeng No. 1’) were employed as the susceptible host. The average lesion length caused by the wild-type *Xcc* 8004 strain on a cabbage leaf was 2.1 ± 0.7 cm at 14 dpi. The *Xcc madB* deletion strain induced a significantly reduced average lesion length (1.1 ± 0.5 cm), whereas the complementary strain *Xcc* Δ*madB::madB* exhibited an average lesion length of 2.3 ± 0.6 cm, not significantly different from that of the wild-type (Fig. 5B). These results collectively indicate that *madB* is required for the pathogenicity of both *Xoo* and *Xcc* in their respective host plants.

### *madB* is involved in the production of virulence-related factors in both *Xoo* and *Xcc*

To further investigate the role of *madB* in virulence, we assessed several pathogenicity-related virulence factors produced by *Xoo* and *Xcc* strains. Initially, we analyzed the extracellular enzymes secreted by both pathogens. The production of extracellular enzymes, particularly cellulase and xylanase, by *Xoo* strains did not exhibit significant differences between the Δ*madB* mutant and the wild-type strain (Fig. S4A). Similarly, the activities of extracellular enzymes, including cellulase, amylase, and protease, in the *Xcc* wild-type strain and the *Xcc* Δ*madB* mutant showed no notable variations (Fig. S4).

**Fig 6:**
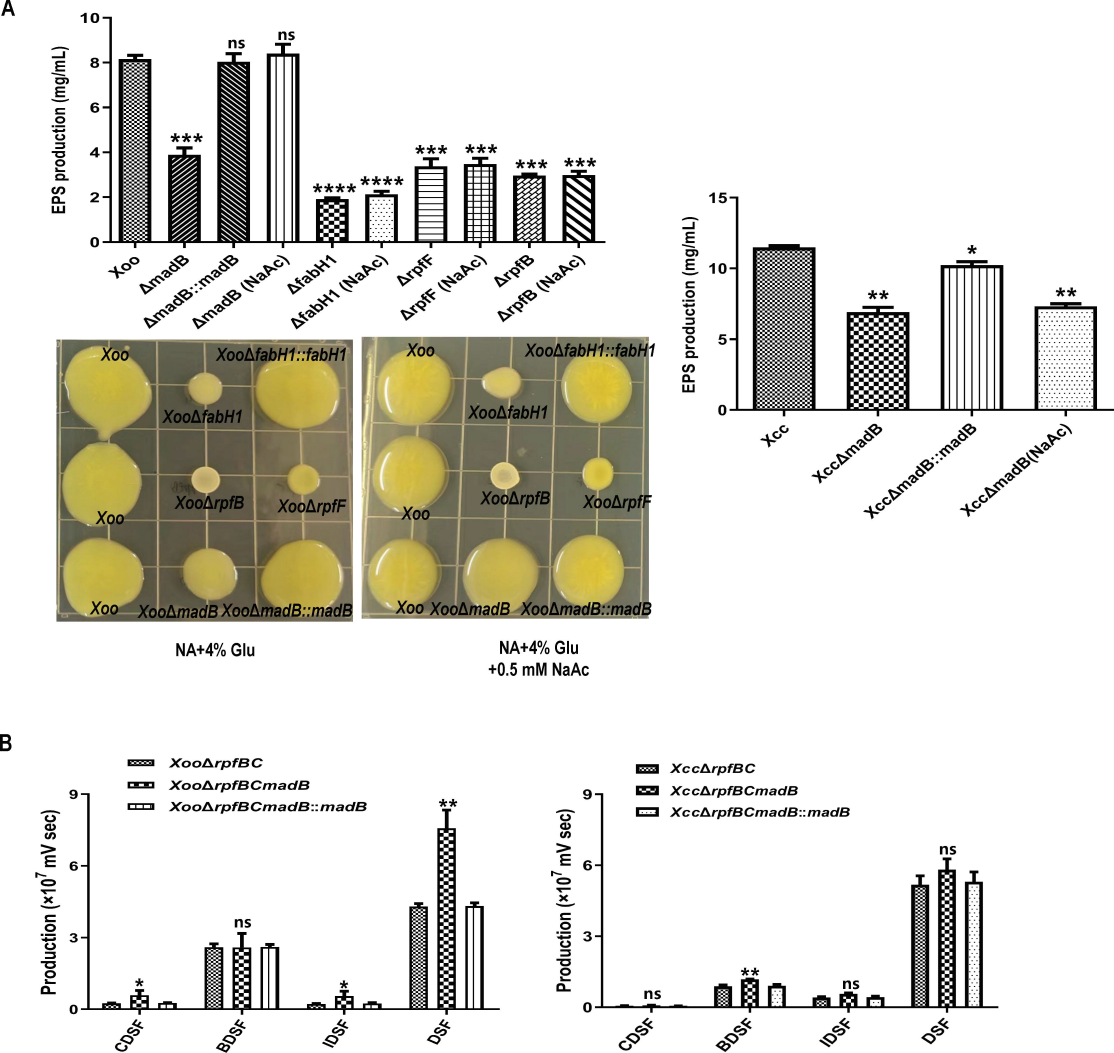
Production of extracellular polysaccharide (EPS) and DSF-family signals in *madB* mutant strains. (**A**) Quantitative determination of EPS produced in madB mutant strains. Sodium acetate was added to NA (*Xoo*) or NYG medium (*Xcc*) at a final concentration of 0.5 mM. The values are presented as the mean ± SD based on three independent experiments. The asterisks above the error bars indicate significant differences compared with the wild-type strain (*t* test, ^***^P < 0.001, ^****^P < 0.0001). (**B**) The production of DSF-family signal in *madB* mutant strains. The levels of the DSF-family signal were calculated based on the peak areas of HPLC analysis. The values are presented as the mean ± standard deviation (SD) from three independent experiments. The asterisks above the error bars indicate significant differences compared with the wild-type strain (*P < 0.05, **P < 0.01).

Next, we examined the production of extracellular polysaccharide (EPS) by mutant strains. The *Xoo* PXO99A strain, the *Xoo* Δ*madB* mutant, and the *Xoo* Δ*madB::madB* strain produced EPS at levels of 8.2 ± 0.3, 3.9 ± 0.5, and 8.0 ± 0.6 mg/mL, respectively (Fig. 6A). Notably, the EPS production by the *Xoo* Δ*madB* mutant was significantly lower compared with the wild-type strain PXO99A, whereas the *Xoo* Δ*madB::madB* strain exhibited no statistically significant difference in EPS production compared with the wild-type strain (Fig. 6A). Additionally, the *Xcc* wild-type strain 8004 produced 11.5 ± 0.2 mg/mL EPS, whereas the *Xcc* Δ*madB* mutant yielded only 6.9 ± 0.4 mg/mL EPS, indicating a significant reduction compared with the wild-type strain (Fig. 6B). These findings suggest that the *madB* gene plays a role in EPS production in both *Xcc* and *Xoo* strains.

UDP-N-acetylglucosamine (UDP-GlcNAc) is a crucial substrate required to produce extracellular polysaccharides (EPS) (15, 39). It has been established that the acetyl group in UDP-GlcNAc is derived from acetyl-CoA or acetyl-ACP (15, 39). Consequently, we hypothesized that the reduced production of acetyl-ACP in *madB* mutants could account for the diminished EPS production in both *Xoo* and *Xcc* strains. To test this, we supplemented the EPS production medium with 0.5 mM sodium acetate and analyzed the EPS production of *Xoo* and *Xcc* strains. As anticipated, the addition of 0.5 mM sodium acetate to the NA medium restored the EPS production of the *Xoo madB* mutant to levels comparable with the wild-type strain (Fig. 6A, left). We also assessed the EPS production in *Xoo rpfF* and *rpfB* mutant strains using the same method. However, sodium acetate failed to restore EPS production in these mutant strains (Fig. 6A, left), indicating that the reduced production of acetyl-ACP in the *Xoo madB* mutant directly caused the decrease in EPS. Interestingly, supplementing sodium acetate did not restore EPS production in the *Xcc madB* mutant, suggesting that *madB* in *Xcc* does not directly influence EPS production (Fig. 6A, right). The underlying mechanism for the decreased EPS production in the *Xcc madB* mutant remains to be further elucidated.

We also monitored biofilm formation by the *Xoo* and *Xcc madB* mutant strains using the crystal violet assay. The results showed that the biofilm biomass produced by the *Xoo madB* mutant increased by 39.5% compared with that of the wild-type strain PXO99A (Fig. S5A). In contrast, the *Xcc madB* mutant exhibited a 22.8% reduction in biofilm biomass compared with the wild-type strain 8004 (Fig. S5A). Furthermore, we assessed the motility of the *madB* mutant strains on semisolid agar, finding that the mutation of *madB* did not significantly affect the motility of either *Xoo* or *Xcc* strains (Fig. S5B).

*Xanthomonas* species utilize quorum sensing (QS) mediated by DSF-family signals to regulate the expression of virulence-contributing factors (18, 22). Consequently, DSF family signals were analyzed in Δ*rpfB*Δ*rpfC*Δ*madB* triple mutants using high-performance liquid chromatography (HPLC) to investigate the effects of MadB on DSF family signal production in both *Xoo* and *Xcc*. Compared with the Δ*rpfB*Δ*rpfC* double mutant*,* the Δ*rpfB*Δ*rpfC*Δ*madB* mutants of both *Xoo* and *Xcc* exhibited an overall increase in DSF family signals production (Fig. 6B). Notably, the *Xoo* Δ*rpfB*Δ*rpfC*Δ*madB* mutant demonstrated a significant increase (Fig. 6B, left), whereas the *Xcc* Δ*rpfB*Δ*rpfC*Δ*madB* mutant showed only a slight increase (Fig. 6B, right). Additionally, the mutation of *madB* in the *Xoo* Δ*rpfB*Δ*rpfC* strain led to a marked increase in branched-chain DSF family signals, including DSF, IDSF, and CDSF (Fig. 6B, left). In contrast, the mutation of *madB* in the *Xcc* Δ*rpfB*Δ*rpfC* mutant significantly elevated the production of the straight-chain DSF-family signal, BDSF (Fig. 6B, right).

### MadB displays distinct functions in fatty acid synthesis within both *Xoo* and *Xcc*

MadB mediates a novel initiation pathway for fatty acid synthesis (15, 17). To elucidate its role in fatty acid synthesis (FAS), the fatty acid composition of membrane lipids in the *Xoo madB* and *Xcc madB* mutants was analyzed. Notably, the fatty acid profiles of the *Xoo* Δ*madB* mutant differed significantly from those of the wild-type strain, *Xoo* PXO99A (Table 1). In the wild-type strain, the straight-chain fatty acids predominantly comprised n-C_16:0_ (39.47% ± 2.2%) and n-C_16:1_ (29.29% ± 2.72%), whereas the branched-chain fatty acids were primarily composed of *iso*-C_15:0_ (7.32% ± 0.05%) and *iso*-C_17:0_ (8.27% ± 0.84%). Following the *madB* mutation, significant reductions in straight-chain fatty acids were observed, with n-C_16:0_ decreasing to 29.39% ± 2.47% and n-C_16:1_ to 21.7% ± 2.99%. Conversely, branched-chain fatty acids increased notably, with *iso*-C_15:0_ rising to 16.21% ± 0.95% and *iso*-C_17:0_ to 15.41% ± 0.72%.

**TABLE 1:** Fatty acid compositions of total lipid extracts from *Xoo* mutant strains^*a*^

Fatty acid^*b*^	Percentage of the total fatty acids		
	*Xoo*	ΔmadB**^c^	Δ*madB*::*madB*
n-C_14:0_	1.19 ± 0.15	1.08 ± 0.09	1.53 ± 0.23
3-OH-C_14:0_	5.21 ± 3.0	8.43 ± 0.17	4.45 ± 1.74
*iso*-C_15:0_	7.32 ± 0.05	16.21 ± 0.95	5.49 ± 0.88
n-C_16:0_	39.47 ± 2.2	29.39 ± 2.47	41.01 ± 2.2
n-C_16:1_	29.29 ± 2.72	21.7 ± 2.99	34.51 ± 2.38
*iso*-C_17:0_	8.27 ± 0.84	15.41 ± 0.72	5.38 ± 1.67
n-C_18:0_	7.87 ± 1.11	6.83 ± 0.39	6.13 ± 2.3
n-C_18:1_	1.38 ± 0.09	0.95 ± 0.65	1.5 ± 0.3

^*a*^ Cells were grown in NA medium at 30°C for 36 h. The total lipids were extracted and trans-esterified to obtain fatty acid methyl esters, and the products were identified using GC-MS. The values are percentages of total fatty acids and are presented as the mean ± standard deviation of three independent experiments.

^*b*^ n-C_14:0_, myristic acid; 3-OH-C_14:0_, 3-hydroxytetradecanoic acid; *iso*-C_15:0_, 13-methyl-tetradecanoic acid; n-C_16:0_, palmitic acid; n-C_16:1_, palmitoleic acid; *iso*-C_17:0_, 15-methyl-palmitic acid; n-C_18:0_, octadecenoic acid; n-C_18:1_, *cis*-11-octadecenoic acid.

^*c*^ Pairwise comparisons were made between the Xoo wild-type strain PXO99A and mutant strains or complemented strains, by Student’s *t*-test (**P < 0.01).

In contrast, the *Xcc* Δ*madB* mutant demonstrated a more subtle alteration in its fatty acid composition compared with the wild-type strain, *Xcc* 8004. There was only a 6% increase in total branched-chain fatty acids, mainly comprising *iso*-C_15:0_ and *anteiso*-C_15:0_ (Table 2). These findings imply that while MadB is indeed involved in FAS, its role varies between *Xoo* and *Xcc*. In *Xoo*, the FAS pathway mediated by MadB operates independently of the pathway controlled by FabH. Consequently, *Xoo* retains the ability to perform *de novo* FAS and sustains bacterial growth even when either *madB* or *fabH* is mutated. Conversely, in *Xcc*, the FabH-mediated pathway is the primary route for FAS, whereas the MadB-facilitated pathway assumes a more regulatory function. Thus, the *fabH* gene in *Xcc* is crucial, and mutations in *madB* have only minor effects on the fatty acid composition of *Xcc*.

**TABLE 2:** Fatty acid compositions of total lipid extracts from *Xcc* mutant strains^*a*^

Fatty acid^*b*^	Percentage of the total fatty acids		
	*Xcc*	Δ*madB*	Δ*madB*::*madB*
*iso*-C_14:0_	0.66 ± 0.33	0.48 ± 0.01	0.72 ± 0.12
n-C_14:0_	3.56 ± 0.27	2.54 ± 0.13	3.91 ± 0.15
*iso*-C_15:0_	15.18 ± 0.25	19.21 ± 1.04	15.8 ± 0.43
*anteiso*-C_15:0_	15.90 ± 0.01	17.92 ± 1.46	14.84 ± 0.29
n-C_15:0_	5.37 ± 1.52	5.49 ± 0.63	6.06 ± 0.51
*iso*-C_16:0_	1.92 ± 0.48	1.65 ± 0.14	2.43 ± 0.02
n-C_16:1_	26.90 ± 0.83	24.83 ± 0.39	26.05 ± 0.47
n-C_16:0_	19.82 ± 0.18	18.75 ± 1.72	19.52 ± 0.43
*iso*-C_17:0_	2.85 ± 0.52	2.75 ± 0.33	2.71 ± 0.41
*anteiso*-C_17:0_	0.65 ± 0.08	0.52 ± 0.02	0.68 ± 0.02
n-C_17:1_	2.11 ± 0.41	1.98 ± 0.18	2.3 ± 0.13
n-C_18:1_	1.73 ± 0.07	1.35 ± 0.54	1.86 ± 0.06
n-C_18:0_	3.36 ± 1.82	2.53 ± 0.80	3.12 ± 0.4

^*a*^ Cells were grown in NYG medium at 30°C for 36 h. The total lipids were extracted and trans-esterified to obtain fatty acid methyl esters, and the products were identified using GC-MS. The values are percentages of total fatty acids and are presented as the mean ± standard deviation of three independent experiments.

^*b*^ *iso*-C_14:0_, 12-methyl tridecanoic acid; n-C_14:0_, myristic acid; *iso*-C_15:0_, 13-methyl-tetradecanoic acid; anteiso-C_15:0_, 12-methyl-tetradecanoic acid; n-C_15:0_, pentadecanoic acid; *iso*-C_16:0_, 14-methyl-pentadecanoic acid; *iso*-C_17:0_, 15-methyl-palmitic acid; n-C_16:0_, palmitic acid; n-C_16:1_, palmitoleic acid; *anteiso*-C_17:0_, 14-methyl-palmitic acid; n-C_17:1_, *cis*-10, *cis*-10-hexadecenoic acid; n-C_18:0_, octadecenoic acid; n-C_18:1_, *cis*-11-octadecenoic acid.

### *madB* exhibits distinct genetic characteristics in *Xoo* and *Xcc*

**Fig 7:**
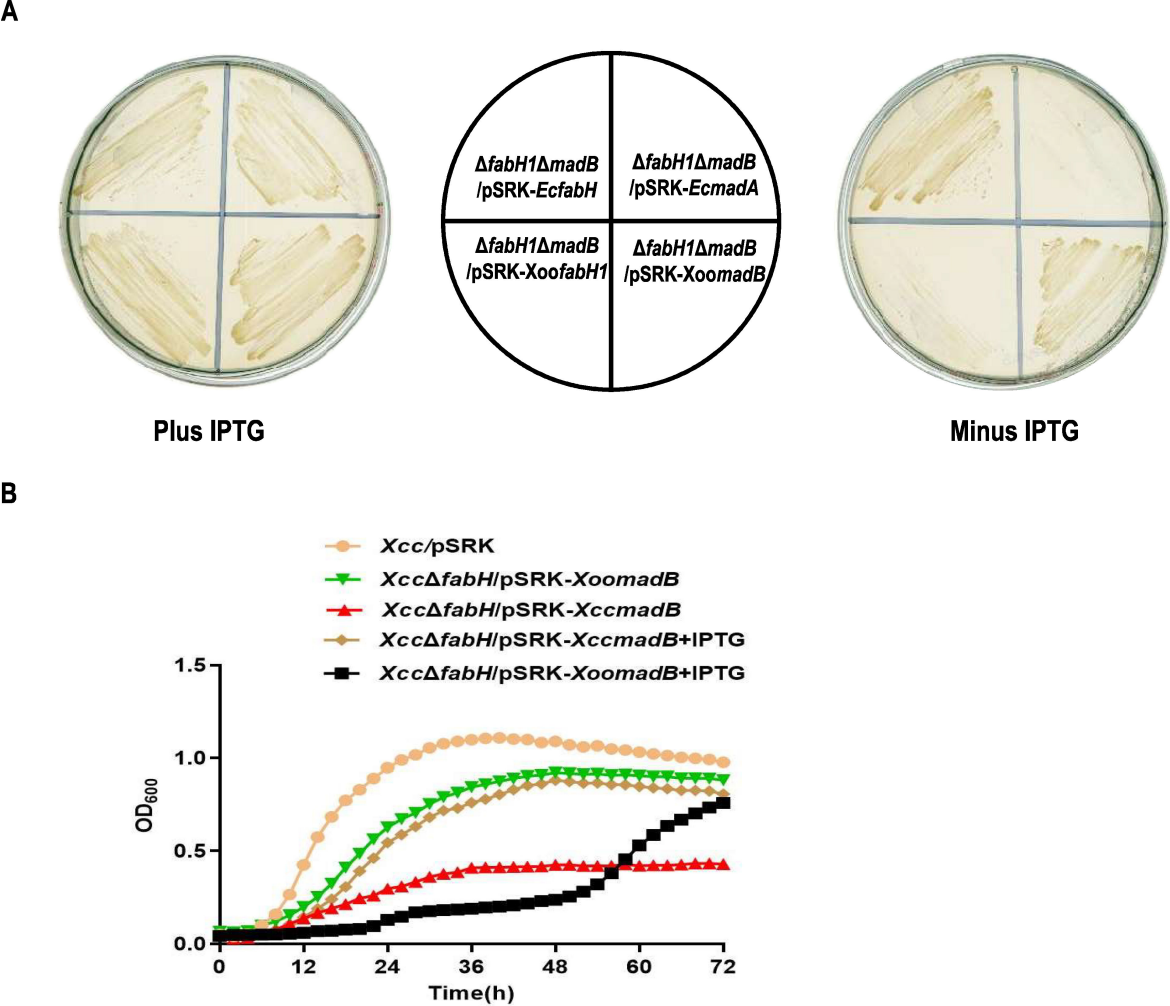
Genetic analysis of the *madB* gene in *Xoo* and *Xcc*. (**A**) Deletion of *madB* and *fabH1* in *Xoo*. (**B**) Overexpression of *madB* restored the growth defect caused by *fabH* mutation in *Xcc*.

To substantiate the above hypothesis, the genetic characteristics of *madB* were examined in both organisms. Initially, we attempted to construct a double-mutant strain, *Xoo* Δ*fabH1*Δ*madB*. After multiple independent experiments, no Δ*fabH1*Δ*madB* double mutant was identified. This outcome suggests that *madB* and *fabH1* cannot be deleted simultaneously, implying the absence of an alternative fatty acid synthesis (FAS) initiation pathway. To validate these findings, we constructed the plasmid pSRK-EcmadA carrying *E. coli madA* gene and introduced it into the *Xoo fabH1* mutant to screen for the double mutant *Xoo* Δ*fabH1*Δ*madB*/pSRK-EcmadA. The mutant Δ*fabH1*Δ*madB*/pSRK-EcmadA was successfully obtained under IPTG induction (Fig. 7A). However, the strain failed to grow without IPTG induction. Similarly, using comparable techniques, we generated *Xoo* Δ*fabH1*Δ*madB*/pSRK-XoomadB and *Xoo* Δ*fabH1*Δ*madB*/pSRK-XoofabH1 strains. Growth analysis indicated that Δ*fabH1*Δ*madB*/pSRK-XoomadB and Δ*fabH*1Δ*madB*/pSRK-XoofabH1 could grow normally under IPTG induction, but Δ*fabH1*Δ*madB*/pSRK-XoofabH1 was unable to grow without IPTG induction (Fig. 7A). These results further corroborate that in *Xoo*, the MadB-mediated initiation pathway of FAS is independent of the FabH-mediated pathway, and *madB* and *fabH1* cannot be deleted simultaneously.

The inability to knock out the *fabH* gene in *Xcc* (28), along with evidence showing that *Xcc madB* can compensate for the growth defect caused by a *fabH* mutation in *R. solanacearum*, raises the question of whether the overexpression of *madB* could functionally replace *fabH* in *Xcc*. To investigate this, the plasmid pSRK-XccmadB was introduced into the wild-type strain *Xcc* 8004, and the mutant strain Δ*fabH*/pSRK-XccmadB was screened under IPTG induction. Without IPTG induction, the strain Δ*fabH*/pSRK-XccmadB exhibited extremely slow growth (Fig. 7B). However, under IPTG induction, the growth of this strain was partially restored, although it remained weaker compared with the wild-type strain *Xcc* 8004 (Fig. 7B). This suggests that enhancing the activity of MadB can partially compensate for the loss of FabH function.

*In vitro* activity analysis confirmed that *Xoo* MadB exhibits higher activity compared with *Xcc* MadB. Based on this, we introduced the plasmid pSRK-XoomadB into *Xcc* 8004 and subsequently isolated the mutant strain Δ*fabH*/pSRK-XoomadB. Interestingly, strain Δ*fabH*/pSRK-XoomadB achieved the same level of growth as Δ*fabH*/pSRK-XccmadB with IPTG induction, even in the absence of IPTG (Fig. 7B). However, under IPTG induction, the growth of Δ*fabH*/pSRK-XoomadB was severely inhibited (Fig. 7B). These findings confirm that although enhanced activity of MadB can partially compensate for the loss of FabH function in *Xcc*, overexpression of MadB, especially with higher activity variants like *Xoo* MadB, may have detrimental effects on growth. This observation further supports the notion that the FabH-mediated pathway serves as the principal route for fatty acid synthesis in *Xcc*. Despite exhibiting some overlapping functions, MadB is incapable of functionally replacing FabH in FAS.

### *madB* exhibits distinct gene arrangement between *Xoo* and *Xcc*

**Fig 8:**
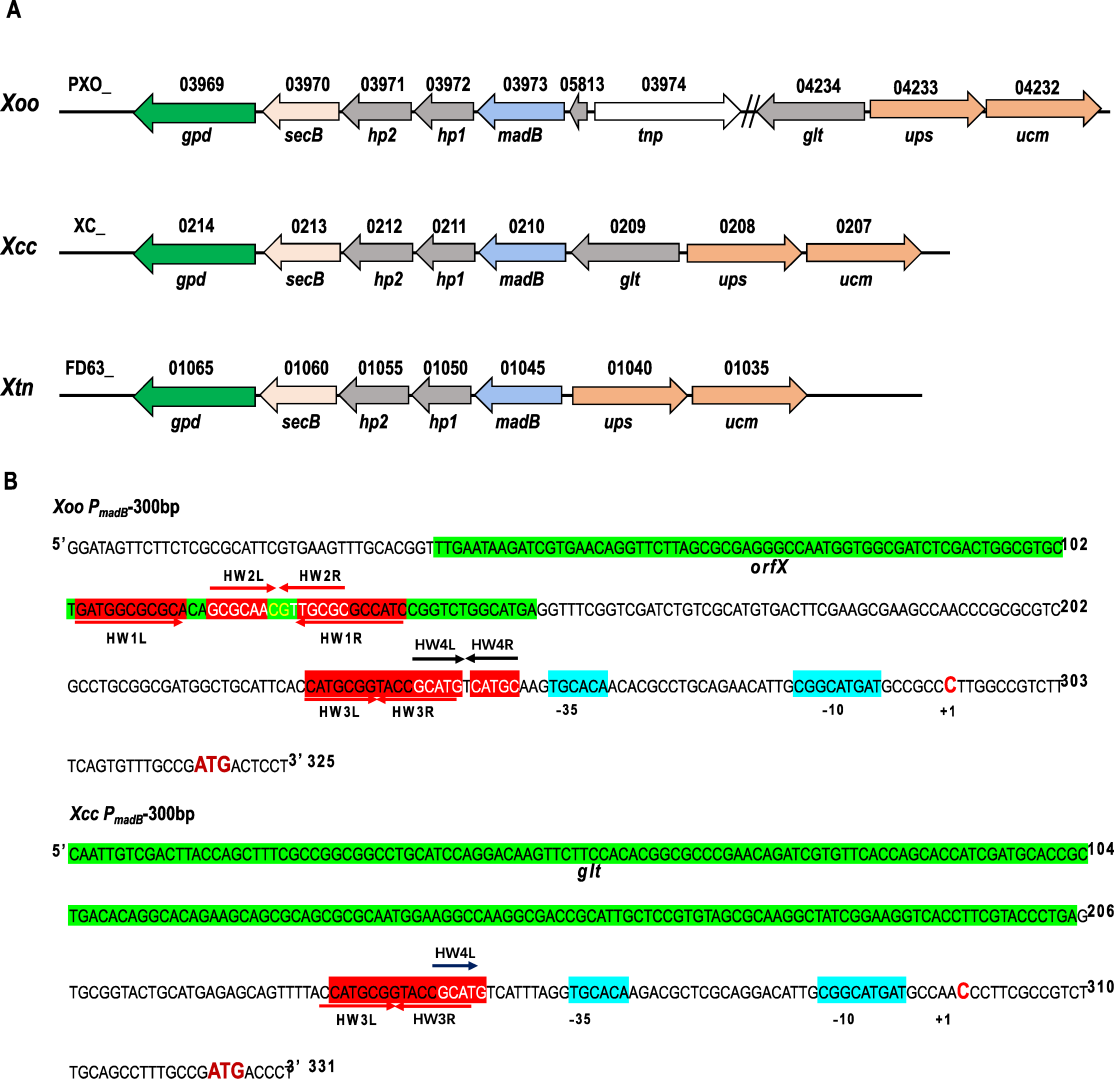
Genomic organization and regulatory regions of *madB* in various *Xanthomonas* species. (**A**) The genomic arrangement of *madB* and its adjacent genes across diverse *Xanthomonas* bacteria. Abbreviations: *glt*, glycosyltransferase; *hp1*, conserved hypothetical protein 1; *hp2*, conserved hypothetical protein 2; *secB*, preprotein translocase subunit SecB; *gpd*, glycerol-3-phosphate dehydrogenase; *ups*, uroporphyrinogen-III synthase; *ucm*, uroporphyrin-III C-methyltransferase; *tnp*, ISXo5 transposase; Xtn, *X. translucens* pv. *undulosa*. (**B**) Bioinformatics analysis of the expression regulation region of *madB*. Abbreviations: *orfX*, open reading frame X; *glt*, glycosyltransferase; HW1, palindromic sequences 1; HW2, palindromic sequences 2; HW3, palindromic sequences 3; HW4, palindromic sequences 4; ATG, start codon; C, the transcription start site (+1).

To gain deeper insights into the divergent functions of MadB in *Xoo* and *Xcc*, we examined the genomic organization of the *madB* genes. Employing *Xoo* MadB as the query, we performed a BLAST search against the genomes of various *Xanthomonas* species available on the KEGG database. Our findings revealed that MadB is widely distributed and highly conserved across different *Xanthomonas* species. The arrangement of *madB* and neighboring genes in these bacterial chromosomes also exhibited significant similarities (Fig. 8A). The primary genes flanking *madB* include *glt* (glycosyltransferase), *hp1* (conserved hypothetical protein 1), *hp2* (conserved hypothetical protein 2), *secB* (preprotein translocase subunit SecB)*, gpd* (glycerol-3-phosphate dehydrogenase), *ups* (uroporphyrinogen-III synthase), and *ucm* (uroporphyrin-III C-methyltransferase). In *Xcc*, *madB* and its neighboring genes were intact and orderly arranged. Specifically, *glt*, *madB*, *hp1*, *hp2*, *secB*, and *gpd* formed a gene cluster (the *mad* cluster), whereas *ups* and *ucm* constituted another gene cluster, transcribing in the opposite direction to the *mad* cluster (Fig. 8A). This identical gene organization was observed not only in *Xcc* but also in *X. vesicatoria*, *X. vasicola* pv. *vasculorum*, *X*. pv. *phaseoli*, *X. perforans*, *X. hortorum*, *X. hortorum* pv. *gardneri*, *X. citri* pv. *citri*, *X. citri* subsp. *citri*, *X. oryzae* pv. *oryzicola*, *X. axonopodis* pv. *citrumelo*, *X. arboricola*, *X. axonopodi*, and *X*. sp. ISO98C4. In the *mad* gene cluster of *X. translucens* pv. *undulosa* (*Xtn*), the *glt* gene was absent, but the arrangement of the remaining genes mirrored that in *Xcc* (Fig. 8A). This specific gene organization was also seen in *X. sacchari*, *X. hyacinthi*, *X. fragariae*, and *X. albilineans*. However, the arrangement of *madB* and its adjacent genes in *Xoo* deviated from the previously mentioned patterns (Fig. 8A). A substantial DNA fragment was inserted between *madB* and *glt*, resulting in the distant separation of *madB* from *glt*, *ups*, and *ucm*. Additionally, a transposase gene (*tnp*; ISXo5 transposase) was located upstream of *madB*, suggesting that the DNA insertion was likely caused by transposons (Fig. 8A). This distinctive arrangement of *madB* was uniquely found in *Xoo*, implying that *madB* has undergone a distinct evolutionary pathway in *Xoo* compared with other *Xanthomonas*.

Utilizing bioinformatics, we examined the 300 bp DNA fragment located upstream of the start codon of the *madB* gene. In *Xoo*, this fragment encompassed the intergenic region between *madB* and *orfX* (167 bp), the reading frame of *orfX* (120 bp), and an additional 29 bp upstream sequence (Fig. 8B, above). Within this 300 bp DNA sequence, positions −32 bp to −40 bp correspond to the −10 region, −60 bp to −65 bp align with the −35 region, −75 bp to −90 bp with the palindromic sequence (HW3), and −69 bp to −79 bp with the palindromic sequence HW4. Additionally, palindromic sequences HW2 and HW1 are, respectively, located from −181 bp to −213 bp and −187 bp to −200 bp upstream of ATG (Fig. 8B, above). In *Xcc*, this fragment included the intergenic region between *madB* and *glt* (119 bp) and the 3' DNA fragment of *glt* (204 bp) (Fig. 8B, below). Within this 300 bp DNA sequence, positions −31 bp to −41 bp correspond to the −10 region, −61 bp to −66 bp to the −35 region, and −76 bp to −91 bp to the palindromic sequence (HW3) (Fig. 8B, below). Notably, *Xcc* lacks the palindromic sequences HW1 and HW2, and HW4 is represented only by HW4L (Fig. 8B, below). DNA sequence alignment revealed that the 95 bp DNA sequences upstream of the start codon in 300 bp DNA fragments are highly homologous, achieving 85% identity (Fig. S6). It is noteworthy that the −10 region, −35 region, and the palindromic sequence HW3 are identical in both DNA fragments. This observation further indicates that *madB* exhibits differential expression in *Xoo* and *Xcc*.

### *madB* exhibits distinct gene expression regulation between *Xoo* and *Xcc*

We performed a preliminary analysis of MadB expression in *Xoo* and *Xcc*. To determine whether *madB* is co-transcribed with its upstream genes, reverse transcription PCR (RT-PCR) was conducted. The results indicated that *madB* does not co-transcribe with *orfX* in *Xoo* nor with *glt* in *Xcc*, suggesting that *madB* is independently transcribed in both *Xoo* and *Xcc* (Fig. S7).

Transcription starts site analysis revealed that in *Xoo*, the transcription start site (+1) of *madB* is located 25 bp upstream of the start codon ATG, whereas in *Xcc*, the transcription start site (+1) of *madB* is situated 26 bp upstream of the start codon ATG (Fig. 8B).

**Fig 9:**
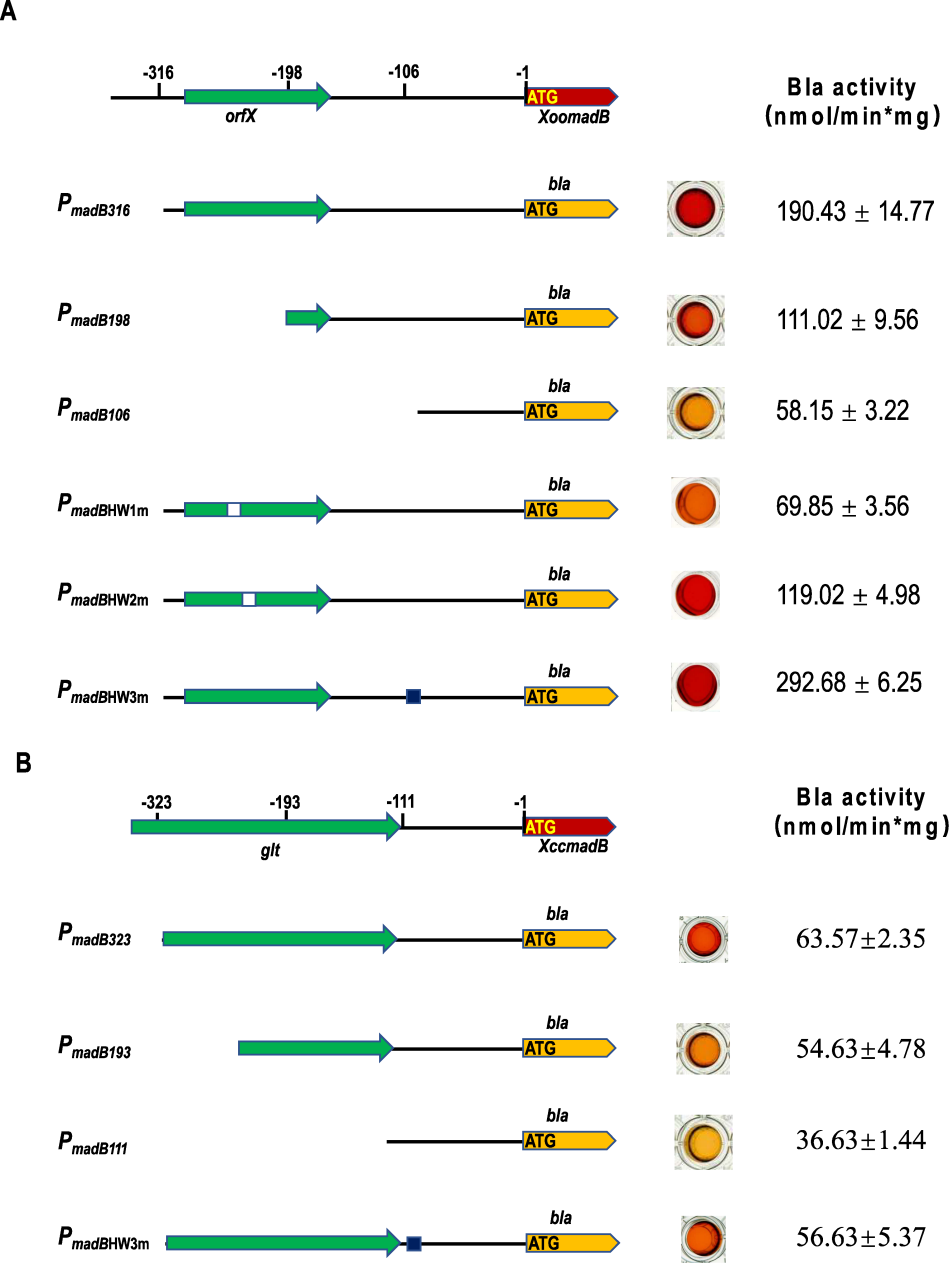
Analysis of *madB* gene expression. (**A**) The expression of *madB* in *Xoo* strain. (**B**) The expression of *madB* in *Xoo* strain. *bla*: the β-lactamase.

The expression of *madB* in *Xoo* and *Xcc* was analyzed using the β-lactamase reporter system. A 300 bp DNA fragment upstream of the *madB* gene was cloned into the plasmid pFA2-Gm-Bla-Flag, which carries the promoter-less *bla* gene, thereby allowing the expression of the *bla* gene to be regulated by this DNA fragment. The recombinant plasmid was then subsequently introduced into *Xoo* or *Xcc*, and β-lactamase activity of bacteria was measured. The results showed that when the recombinant plasmid carrying the 300 bp DNA fragment upstream of the *Xoo madB* gene was introduced into the wild-type *Xoo* strain, the β-lactamase activity was 190.43 ± 14.77 nmol/min per mg (Fig. 9A). In contrast, when the recombinant plasmid carrying the 300 bp DNA fragment upstream of the *Xcc madB* gene was introduced into the *Xcc* strain, the β-lactamase activity was significantly reduced to 63.57 ± 2.35 nmol/min per mg (Fig. 9B). Additionally, we truncated the 300 bp DNA fragment from the 5' end to construct new recombinant plasmids, which were introduced into *Xoo* or *Xcc*, and β-lactamase activity was measured again. The results showed that when *Xoo*’s 300 bp DNA fragment was truncated, β-lactamase activity in the bacteria decreased sharply (Fig. 9A), whereas the truncation of the 300 bp DNA fragment of *Xcc* led to a less significant decrease in β-lactamase activity (Fig. 9B). These findings indicate that *madB* expression in *Xoo* is strictly regulated, whereas in *Xcc*, *madB* is constitutively expressed.

To confirm this hypothesis, we mutated the palindromic sequences within the 300 bp DNA fragment. The results showed that mutating the palindromic sequence HW1 in *Xoo*’s 300 bp DNA fragment significantly reduced β-lactamase activity, and mutating the palindromic sequence HW2 also decreased β-lactamase activity to a certain extent (Fig. 9A). However, after mutating the palindromic sequence HW3, β-lactamase activity significantly increased (Fig. 9A). In contrast, mutating the palindromic sequence HW3 in *Xcc*’s 300 bp DNA fragment did not significantly alter β-lactamase activity (Fig. 9B). These findings strongly indicate that *madB* expression in *Xoo* is regulated by multiple mechanisms, whereas the regulation of *madB* expression in *Xcc* is relatively simple.

## DISCUSSION

Mad was originally identified in the *fabH* mutant of *E. coli* (15, 16), where it catalyzed the decarboxylation of malonyl-ACP to acetyl-ACP, facilitating an alternative, FabH-independent initiation pathway in bacterial FAS (15, 17). Two distinct types of Mad proteins have been characterized. In *E. coli*, the Mad protein is encoded by *madA* and contains an amino-terminal N-acetyl transferase domain fused to a carboxy-terminal hot dog-fold domain (YiiD_C domain), responsible for the malonyl-ACP decarboxylase activity (15). In contrast, in *P. putida*, the Mad protein is encoded by *madB* and comprises a standalone YiiD_C domain responsible for malonyl-ACP decarboxylation (17). Despite the high conservation of the *mad* gene among Proteobacteria, the physiological functions of the Mad protein remain elusive (15, 16). Some propose that Mad proteins contribute to the homeostatic regulation of acetyl CoA *in vivo*, whereas others suggest that primary supply acetyl groups for the synthesis of UDP-GlcNAc (15). Further research is needed to resolve these questions.

Both *Xoo* and *Xcc* encode MadB, exhibiting 87.5% sequence identity between their MadB proteins. These *madB* genes not only genetically compensated for the growth defect in a *R. solanacearum fabH* mutant but also restored the growth in an *E. coli fabH madA* double mutant. *In vitro*, both MadB proteins catalyzed the decarboxylation of malonyl-ACP to acetyl-ACP. These data collectively indicate that the two MadBs possess identical enzymatic properties.

However, although the single mutation of *madB* reduced the pathogenicity of *Xoo* and *Xcc* toward their host plants, the *Xoo madB* and *Xcc madB* mutant strains exhibited numerous distinct phenotypic traits. These phenotypic distinctions manifest primarily in the following four aspects. (i) The deletion of *madB* in *Xoo* slightly curtails its growth while having a negligible impact on its environmental stress tolerance. In *Xcc*, the deletion of *madB* does not substantially alter *Xcc* growth but markedly enhances susceptibility to various environmental stresses. (ii) The mutation of *madB* reduces the production of EPS in both bacteria; however, acetate supplementation can reinstate EPS production in *Xoo madB* strains, whereas it fails to do so in *Xcc madB* mutant strains. (iii) The mutation of *madB* leads to a substantial increase in the synthesis of branched-chain DSF-family signals in *Xoo*, whereas in *Xcc*, only a marginal augmentation of BDSF signals occurs. (iv) The mutation of *madB* results in a significant increase in the proportion of branched-chain fatty acids in the membrane lipids of *Xoo* mutant strains, whereas the fatty acid composition of membrane lipids in *Xcc madB* strains remains relatively unchanged. These observations underscore the significant divergence in the physiological role of *madB* between *Xoo* and *Xcc*.

Biochemical and genetic analyses have revealed that the underlying cause of these disparate physiological roles is the distinct biochemical functions of MadB in the two bacteria. In *Xoo*, the FAS pathway mediated by MadB operates independently of the FAS pathway mediated by FabH. Consequently, the deletion of either *madB* or *fabH* individually still permits bacterial growth. Upon knocking out *madB*, the FAS pathway mediated by FabH becomes the exclusive route for fatty acid synthesis in *Xoo*, leading to a substantial increase in the proportion of branched-chain fatty acids in membrane phospholipids and elevating the content of branched-chain DSF-family signals. Despite this increase in branched-chain fatty acids, it does not disrupt cell membrane permeability, and thus, the bacterium’s response to environmental stress remains unchanged. However, the mutation of *madB* results in a reduced supply of acetyl-ACP, directly impacting EPS synthesis. In *Xcc*, the FAS pathway mediated by FabH is the primary pathway, whereas the MadB pathway plays a secondary and regulatory role. Therefore, in *Xcc*, *fabH* is an essential gene and cannot be deleted. When *madB* is knocked out, fatty acid synthesis in *Xcc* is not greatly affected; hence, the DSF family signals produced by the *madB* mutant do not differ significantly from those of the wild-type strain, and only cause minimal changes in the proportion of membrane phospholipid fatty acids. However, such changes affect membrane permeability, making *madB* mutants more susceptible to environmental stress. Since the MadB pathway primarily exerts regulatory functions, the acetyl-ACP generated by this pathway is limited; hence, it does not directly impact EPS synthesis.

The divergence in MadB’s biochemical function between *Xoo* and *Xcc* may be attributed to genomic evolutions that have adapted to distinct host environments. In the *Xcc* genome, the genes *glt*, *madB*, *hp1*, *hp2, secB*, and *gpd* are grouped together to form the *mad* cluster, whereas *ups* and *ucm* comprise a separate gene cluster. Conversely, in the *Xoo* genome, the organization surrounding *madB* exhibits a markedly different configuration compared to *Xcc*. Specifically, a substantial DNA segment is inserted between *madB* and *glt*, resulting in the significant spatial separation of *madB* from *glt*, *ups,* and *ucm*. Notably, most *Xanthomonas* species exhibit a *madB* arrangement similar to *Xcc*, with only *Xoo* displaying this unique configuration. This unique genomic structure in *Xoo* suggests adaptive strategies tailored to its specific ecological niche.

Furthermore, we analyzed the expression of *madB* and found that its expression in *Xoo* is regulated by multiple complex mechanisms, whereas in *Xcc*, the regulation of *madB* expression is relatively straightforward. This differential regulation further supports the notion that *madB* likely performs distinct physiological functions in these two species.

In summary, although both *Xoo* and *Xcc* have MadB-mediated initiation pathways for fatty acid synthesis, the two pathways perform different physiological functions in *Xoo* and *Xcc*.

## MATERIALS AND METHODS

### Materials

The sources of supplies were as follows: malonyl-CoA, acetyl-CoA, fatty acids, NADPH, NADH, nitrocefin, and antibiotics were procured from Sigma-Aldrich. Molecular biology reagents were provided by Takara Biotechnology (Dalian) Co., Ltd. Ni-NTA agarose columns were acquired from Invitrogen (Shanghai), whereas the Quick Start Bradford dye reagent was supplied by Bio-Rad (America). HC-C_18_ HPLC columns were provided by Agilent Technologies (Palo Alto, CA, United States). Oligonucleotide primers were synthesized by Sangon Biotech (Shanghai) Co., Ltd. All other chemicals and reagents used were of the highest available quality.

### Bacterial strains, plasmids, and bacterial growth conditions

The bacterial strains and plasmids utilized are detailed in Table S1. *E. coli* cultures were cultivated at 37°C in LB medium (yeast extract, 5 g/L; peptone, 10 g/L; NaCl, 10 g/L; pH 7.0). *R. solanacearum* and its derived mutants were cultivated at 30°C in BG medium (peptone, 10 g/L; yeast extract, 1 g/L; casamino acids, 1 g/L; glucose, 5 g/L; pH 7.0) (31). *X. oryzae* pv. *oryzae* (*Xoo*) and its derived mutants were cultured at 30°C in NA medium (beef extract, 3 g/L; yeast extract, 1 g/L; peptone, 5 g/L; sucrose, 10 g/L; pH 7.0) (40). *X. campestris* pv. *campestris* (*Xcc*) and its derived mutants were grown at 30°C in NYG medium (tryptone, 5 g/L; yeast extract, 3 g/L; glycerol, 20 g/L; pH 7.0) (28). When necessary, antibiotics and inducers were added to the cultures as follows: kanamycin sulfate, 30 µg/mL; spectinomycin, 100 µg/mL; gentamicin, 30 µg/mL; tetracycline, 10 µg/mL; sodium ampicillin, 100 µg/mL; chloramphenicol, 30 µg/mL; IPTG, 1 mM. Octanoic acid was employed at a final concentration of 1 mM. The growth of bacteria was assessed either by colony formation on solid media or by measuring the optical density at 600 nm.

### Complementation of the *R*. *solanacearum fabH* deletion strain

The *madB* coding sequences were PCR-amplified from the genomic DNA of *Xoo* PXO99A or *Xcc* 8004 using the primers listed in Table S2. The amplified fragments were then purified and inserted into the multicopy plasmid pSRK-Tc (30) to yield pSRK-XoomadB or pSRK-XccmadB, respectively. These plasmids were subsequently confirmed through nucleotide sequencing conducted by Sangon Biotech (Shanghai) Co., Ltd. Derivatives of *E. coli* strain S17-1, carrying these plasmids, were mated with the *R. solanacearum fabH* deletion strain Δ*RsfabH* on BG plates supplemented with octanoic acid for 48 h at 30°C (31). The resulting cells were suspended in BG medium, and appropriate dilutions were spread onto BG plates containing octanoic acid and chloramphenicol plus tetracycline. The conjugants were then inoculated onto BG plates, with or without octanoic acid, and their growth was assessed after 2 days of incubation at 30°C.

### Complementation of the *E*. *coli fabH madA* temperature-sensitive strain

*E. coli* ∆*fabH*∆*madA*/pRP-EcmadA harbors the temperature-sensitive plasmid pRP-EcmadA, which is derived from the plasmid pRP1028 (41), and carries the *E. coli madA* gene under LacI control. Additionally, pRP-EcmadA contains a gene encoding a red fluorescent protein (41), causing the ∆*fabH*∆*madA*/pRP-EcmadA strain to exhibit red colonies during growth. This strain can eliminate the plasmid pRP-EcmadA and grow at 42°C to form white colonies, provided that the transgene introduced can functionally substitute for the *E. coli fabH* or *madA* genes. Transformants of the temperature-sensitive *E. coli* strain ∆*fabH*∆*madA*/pRP-EcmadA, harboring plasmids pSRK-XoomadB or pSRK-XccmadB, were spread on LB plates containing 1 mM IPTG and 10 µg/mL tetracycline and incubated for 12 h at 42°C. Representative white colonies were subsequently picked and streaked onto fresh plates to confirm their growth at 42°C.

### Expression and purification of proteins

The plasmid pET-28(b), containing the *madB* coding sequence from either *Xoo* or *Xcc*, was transformed into *E. coli* BL21(DE3). The transformants were incubated and harvested, and then, MadBs were purified using a nickel-ion affinity column (Qiagen) according to standard protocols. The purified proteins were monitored using SDS-PAGE. Additionally, *E. coli* proteins FabB, FabH, FabG, FabI, FabA, *apo* and *holo*-ACP, *Vibrio harveyi*’s AasS, and *Bacillus subtilis*’s Sfp proteins were purified as previously described (32, 42).

### Enzymatic synthesis of malonyl-ACP and octanoyl-ACP

The enzymatic synthesis of malonyl-ACP and octanoyl-ACP was carried out as previously described (32, 42). Malonyl-ACP was synthesized from *E. coli apo*-ACP and malonyl-CoA using *B. subtilis* Sfp. The reaction mixtures contained 50 mM Tris-HCl (pH 8.0), 10 mM MgCl_2_, 1 mM β-mercaptoethanol, 100 µM malonyl-CoA, 50 µM *apo*-ACP, and 5 µM *B. subtilis* Sfp, followed by incubation for 1 h at 37°C. The resulting malonyl-ACP was purified as previously described (5).

For the synthesis of octanoyl-ACP (C_8:0_-ACP), octanoic acid and *holo*-ACP were used with *V. harveyi* AasS. The reaction mixture included 50 mM Tris-HCl (pH 8.0), 10 mM MgCl_2_, 1 mM β-mercaptoethanol, 10 mM ATP, 0.5 mM octanoic acid, 25 µM *holo*-ACP, and 5 µM *V*. *harveyi* AasS, followed by incubation for 1 h at 37°C. The resulting octanoyl-ACP was purified as previously described (42).

### Assay of 3-ketoacyl-ACP synthase III and malonyl-ACP decarboxylase activities *in vitro*

The activities of KAS III in the initiation of fatty acid synthesis were evaluated using reaction mixtures composed of 0.1 M sodium phosphate (pH 7.0), 1 mM β-mercaptoethanol, 0.1 µg each of *E. coli* FabG, FabA, and FabI, 50 µM NADPH, 50 µM NADH, 100 µM malonyl-ACP, and 100 µM acetyl-CoA in a final volume of 40 µL (27, 31). The reactions were initiated by adding FabHs to the mixture, followed by incubation for 1 h at 37°C. For assessing the malonyl-ACP decarboxylase activities, reaction mixtures containing 0.1 M sodium phosphate (pH 7.0), 1 mM β-mercaptoethanol, 0.1 µg each of *E. coli* FabB, FabG, FabA, and FabI, 50 µM NADPH, 50 µM NADH, and 100 µM malonyl-ACP in a final volume of 40 µL were used. The reactions were initiated by adding MadB to the mixture, followed by incubation for 1 h at 37°C. The reaction products were analyzed using conformationally sensitive gel electrophoresis on 17.5% polyacrylamide gels containing 0.5 M urea. The gels were stained with InstantBlue Coomassie Protein Stain to visualize the products.

The malonyl-ACP decarboxylase activities of *Xoo* MadB or *Xcc* MadB were determined by monitoring the rate of oxidation of NADPH at 340 nm using an extinction coefficient of 6,220 M^−1^ (31). The reaction mixtures for activity assays contained 200 µM NADPH, 0.1 µg of the purified native FabB, and FabG, 500 µM malonyl-ACP, and 0.1 M sodium phosphate buffer (pH 6.4). The reactions were initiated by the addition of 0.1 µg of MadB.

### Construction of in-frame deletion mutants and complementation

The construction of an in-frame deletion mutant of the *madB* gene in *Xoo* or *Xcc* was performed using the previously described methods (27, 43). The DNA fragments upstream and downstream of the *madB* gene in *Xoo* or *Xcc* were PCR-amplified using the corresponding primer sets (Table S2), respectively. The upstream and downstream fragments were fused and cloned into the vector pK18mobsacB (37). The resulting plasmids were introduced into *Xoo* or *Xcc* using the electroporation method, respectively. Single crossover integrants were selected on plates containing kanamycin and confirmed by PCR. Several single crossover integrant colonies were then inoculated into NA (for *Xoo*) or NYG (for *Xcc*) without kanamycin at 30°C for 36 h. Appropriate dilutions were spread on NA or NYG plates containing 10% sucrose. Colonies sensitive to kanamycin were screened using colony PCR with primers listed in Table S2 to obtain the deletion mutants. The deletion mutants were verified by sequencing. For single-copy complementation of *madB*, the coding regions of *madB* plus its promoter region in *Xoo* or *Xcc* were amplified by PCR and were cloned in a versatile mini-Tn7 delivery vector mini Tn7T-Gm plasmid (44), respectively. The resultant constructions were transferred into *Xoo* or *Xcc* by electroporation with the helper plasmid pTNS2, and the complementation strains were selected as described previously (44).

### Analysis of fatty acid composition

To determine fatty acid composition, the strains were grown in NA medium or NYG medium to an OD_600_ of 0.6. Cells were harvested and washed three times with sterile phosphate buffered saline (PBS) buffer. Fatty acid methyl esters were generated and extracted, and then analyzed by gas chromatography-mass spectrometry (GC-MS) as previously described (28, 42).

### Extraction and purification of DSF family signals

The extraction and purification method has been previously detailed (45). Given that the *Xoo* and *Xcc rpfBC* double-deletion strain produced elevated levels of DSF family signals suitable for analysis, the in-frame deletion of *madB* within this double-deletion strain was executed using a method described earlier (26, 43). To quantify DSF-family signal production in each strain using HPLC, the *Xoo* or *Xcc* strains were incubated in a liquid medium for 24 h, after which 50 mL of the supernatant was collected. The crude ethyl acetate extracts underwent filtration through a 0.45 µm Minisart filter unit and were subsequently concentrated to 0.1 mL for HPLC analysis. A 20 µL aliquot of the extract was injected into a C_18_ reverse-phase HPLC column (4.6 mm × 250 mm, Agilent Technologies), which was eluted with a binary mixture of water and methanol (23:77 vol/vol, 0.1% formic acid) at a flow rate of 1 mL/minute using a Shimadzu prominence LC-20AT system (Shimadzu International Trading Co. Limited) equipped with a UV220 detector. The experiments were conducted in triplicate, yielding consistent results. The relative quantities of signal molecules were determined based on peak areas.

### Effects of environmental factors on the growth of *Xoo* and *Xcc* mutants

The bacterial cells of each strain were grown in either NA medium or NYG medium in a shaking incubator at 30°C for two days. The cells were then washed and resuspended in a sterile PBS buffer to achieve a final OD_600_ of approximately 2.0. Serial 10-fold dilutions of the cells were prepared, and 5 µL of the appropriately diluted cells was spotted onto NA plates or NYG plates supplemented with varying concentrations of NaCl, SDS, pH adjusters, or H_2_O_2_. All plates were incubated at 30°C for three days.

### Analysis of membrane integrity

The membrane permeability was measured using a 1-N-phenylnaphthylamine (NPN) uptake assay as previously described with minor modifications (46). The bacterial cells of each strain were grown in either NA medium or NYG medium in a shaking incubator at 30°C for 2 days. The obtained cells were washed three times and then resuspended in 1.0 mL of 4-(2-hydroxyethyl)-1-piperazineethanesulfonic acid (HEPES) buffer (5 mM, pH 7.2) to an OD_600_ of 0.5. Then, 3.90 mL of bacteria suspension was mixed with 100 µL of NPN solution (400 µM in acetone). After 10 min of incubation at room temperature, the changes in fluorescence were immediately measured with an enzyme-labeled instrument (excitation 350 nm, emission 420 nm), and the results were expressed as relative fluorescence intensity.

### Pathogenicity tests

The virulence of *Xoo* strains was assessed on rice cultivars (*Oryza sativa* L. *japonica* cv. *Nipponbare*), following the method previously detailed (38, 47). Similarly, the virulence of *Xcc* strains against their host cabbage plant (*Brassica oleracea* ‘Jingfeng No. 1’) was evaluated, as described earlier (48). In brief, various bacteria were incubated in NA or NYG medium at 30°C for 2 days, after which the cells were washed and re-suspended in sterile PBS buffer to achieve an OD_600_ of 0.1. The cells were then inoculated into the leaves via leaf-clipping. The length of the lesions was measured 14 days post-inoculation. Each test strain was inoculated onto 20 leaves. Each strain was subjected to testing in a minimum of three independent experiments.

### Swimming and swarming motility assays

Swimming and swarming motility assays were conducted on semi-solid plates containing 0.3% agarose and 0.5% agarose, respectively, as outlined in previously referenced methods (32). For mutant strains, bacterial cells were cultured in NA medium or NYG medium in a shaking incubator at 30°C for 2 days. Post-cultivation, the cells were washed and resuspended in a sterile PBS buffer to achieve a final OD_600_ of 2.0. Aliquots of 2 µL of bacterial cell suspension were then spotted on the plates. The plates were incubated at 30°C for 3 days. These assays were replicated a minimum of three times for each strain.

### Measurement of extracellular enzymatic activity and EPS production

The extracellular enzyme assays were carried out according to previously documented protocols (49, 50). For each *Xoo* strain, 2 µL of the culture (with an OD_600_ ≈ 1.0) was spotted onto NA plates containing 0.5% (wt/vol) carboxymethylcellulose (for evaluating cellulase) or 0.2% xylan (for assessing xylanase). These plates were subsequently incubated at 30°C for 2–3 days. Similarly, 2 µL of each *Xcc* strain culture (with an OD_600_ ≈ 1.0) was spotted onto NYG plates containing 1% (wt/vol) skim milk (for protease), 0.5% (wt/vol) carboxymethylcellulose, or 0.1% (wt/vol) starch (for amylase), followed by incubation at 30°C for 2–3 days.

To visualize the results, carboxymethylcellulose (CMC) plates were stained with a 1% Congo Red solution. Xylan plates and starch plates were stained with an I_2_-KI solution composed of I_2_ at 0.08 mol/L and KI at 3.2 mol/L. The zones of clearance around the inoculation spots were photographed, and the relative activities of the enzymes were inferred from the diameters of the clear zones. Each experimental run was repeated three times with three replicates per strain.

To quantify EPS production, cultures of various *Xoo* or *Xcc* strains (1 mL, OD_600_ ≈ 1.0) were incubated in 50 mL of NA medium or NYG medium supplemented with 4% glucose at 30°C for 4 days, with shaking at 180 rpm. EPS was precipitated from the culture supernatant by adding an equal volume of cold 100% ethanol (43). The precipitated EPS was collected by centrifugation at 12,000 g, 4°C for 15 min, air-dried, and weighed. Each experiment involved inoculating three flasks per strain and was repeated three times. Additionally, EPS production of different strains, as indicated by colony morphology and size, was evaluated using a plate method (38). Aliquots of 5 µL (with an OD_600_ ≈ 2.0) of bacterial cells were spotted onto NA plates containing 4% glucose and incubated at 30°C for 3 days. The plates were then cultured under light for an additional 2–3 days. Sodium acetate was incorporated into the NA or NYG medium at a final concentration of 0.5 mM to facilitate EPS production.

### Biofilm formation assay

Biofilm formation assays were conducted following previously described methods with minor modifications (27). The procedure involved incubating diluted bacterial solutions in borosilicate glass tubes for 7 days at 30°C without shaking. Following this incubation, non-adherent bacteria and medium were carefully removed, and the tubes were washed three times to eliminate residual loose cells. The attached biofilm was then incubated with a 0.1% (wt/vol) crystal violet solution for 30 min at room temperature. After staining, the tubes were thoroughly washed twice with water to remove unbound dye and air-dried. The crystal violet bound to the cells was solubilized in 90% ethanol, and the concentration of the dye was quantified by measuring the absorption at 590 nm. Each biofilm formation assay was repeated a minimum of three times to ensure consistency and reliability of the results.

### 5’RACE and reverse transcription-PCR

Total RNA was extracted and purified from bacterial cells cultured in 3 mL volumes using the E.Z.N.A. Bacterial RNA Kit (Omega Bio-Tek, Inc.). The extracted RNAs were subsequently converted to single-stranded cDNAs using the PrimeScriptTM RT reagent Kit (Takara Bio Inc.). The resulting cDNA served as a template for PCR amplification of the *mad* gene cluster in *Xoo* or *Xcc*, using specific primers as detailed in Table S2. To determine the transcriptional start sites of both *Xoo madB* and *Xcc madB*, the 5′ RACE kit from Sangon Biotech was utilized. Initially, a poly(C) tail was appended to the 3′ end of the cDNA using terminal deoxynucleotide transferase. Subsequently, the modified cDNA was amplified using the RACE adapter primer and the outer primer as detailed in Table S2. Ultimately, the PCR products were sequenced by Sangon Biotech (Shanghai) Co., Ltd.

### β-Lactamase activity assay

A 300 bp DNA fragment upstream of either the *Xcc madB* or *Xoo madB* gene was amplified using the primer sets specified in Table S2. Additionally, we performed truncations on the 300 bp DNA fragment from the 5' end, employing PCR with the primer sets listed in Table S2. To introduce site-specific mutations in the palindromic sequences HW1, HW3, and HW3 within the 300 bp DNA fragment, site-directed mutagenesis was executed using the corresponding primer sets as detailed in Table S2.

All these amplified fragments, including the full-length, truncated, and mutation versions, were then ligated with the promoter-less *bla* gene in the plasmid pFA2-Gm-Bla-Flag, specifically between the *Hind* III and *Xba* I sites. This process yielded various β-lactamase reporter vectors capable of monitoring promoter activity under different conditions. These reporter plasmids were subsequently introduced into wild-type *Xoo* or *Xcc* strains via the electroporation method.

To determine β-lactamase activity, the assay was performed spectrophotometrically at a wavelength of 486 nm (51), using 100 nmol of nitrocefin as the substrate. The reaction mixture was prepared in a total volume of 200 µL. The β-lactamase activity was quantified based on the rate of nitrocefin hydrolysis, which results in a change in absorbance at 486 nm.

### Statistical analyses

ANOVA was conducted on experimental data sets using GraphPad Prism 7.0. Error bars in the figures represent standard deviations (SD) as specified in the figure legends. All data are presented as the mean ± SD from at least three independent experiments. The significance of treatment effects was assessed using F-values, with a significance level of *P* = 0.05. If a significant F-test result was obtained, the means were separated using Fisher’s protected least significant difference (LSD) test at *P* ≤ 0.05.

## Data Availability

The data that support the findings of this study are available from the corresponding author upon reasonable request.

## SUPPLEMENTAL MATERIAL

Supplemental material aem.02436-24-s0001.doc Tables S1 to S3; Figures S1 to S7.

ASM does not own the copyrights to Supplemental Material that may be linked to, or accessed through, an article. The authors have granted ASM a non-exclusive, world-wide license to publish the Supplemental Material files. Please contact the corresponding author directly for reuse.
