# Supplementary material for: Functional disparities of malonyl-ACP decarboxylase between Xanthomonas campestris and Xanthomonas oryzae
Source: Appl Environ Microbiol. 2025 Apr 8;91(5):e02436-24. doi: 10.1128/aem.02436-24 (PMC12093956; doi:10.1128/aem.02436-24)
Supplement: Supplemental material — Tables S1 to S3; Figures S1 to S7. [file aem.02436-24-s0001.doc]

**Functional Disparities of Malonyl-ACP Decarboxylase between *Xanthomonas campestris* and *Xanthomonas oryzae***

^#^Mingfeng Yan^1, 2^, ^#^Yonghong Yu^3^, ^#^Lizhen Luo^2^, Jingtong Su^2^, Jincheng Ma^2^, Zhe Hu^2*^ and Haihong Wang^2*^

^1^Institute of Plant Protection, Jiangxi Academy of Agricultural Sciences, Nanchang, Jiangxi 330000, China

^2^Guangdong provincial key laboratory for the development biology and environmental adaptation of agricultural organisms, College of Life Sciences, South China Agricultural University, Guangzhou, Guangdong 510642, China

^3^Guangdong Food and Drug Vocational College, Guangzhou, Guangdong 510520, China

**Running Title:** Malonyl-ACP Decarboxylase in *Xanthomonas*

^#^Mingfeng Yan, Yonghong Yu and Lizhen Luo contributed equally to this article. The order of their names was determined by flipping a coin.

^*^Address correspondence to Haihong Wang, [wanghh36@scau.edu.cn](mailto:wanghh36@scau.edu.cn) or Zhe Hu, zhehu@scau.edu.cn.

The authors declare no conflict of interest.

**Supplementary Materials**

**Tables**

**Table S1 Bacterial strains and plasmids used in this study.**

| **Strain/Plasmid** | **Relevant characteristics** | **Sources** |
| --- | --- | --- |
| ***Escherichia coli*** | | |
| DH-5α | φ80d *lacZ*ΔM15 *endA1recA1hsdR17* (r_K_^-^, m_K_^+^) | Laboratory stock |
| S17-1 | Tp^r^ Sm^r^*recA, thi, pro, hsdR^-^M^+^* RP4::2- Tc::Mu:Km ::Tn*7, λpir* | Laboratory stock |
| BL21(DE3) | *omp*T *hsd*S B (rB^-^ mB^-^) | Laboratory stock |
| MG1655 | Wild-type | Laboratory stock |
| **Δ***EcfabH* | MG1655 *fabH* deletion mutant | Laboratory stock |
| **Δ***EcfabH*/pSRK-*EcmadA* | **Δ***EcfabH* carrying the plasmid of pSRK-*EcmadA* | This study |
| **Δ***EcfabH*/pSRK-*XoomadB* | **Δ***EcfabH* carrying the plasmid of pSRK-*XoomadB* | This study |
| **Δ***EcfabH*/pSRK-*XccmadB* | **Δ***EcfabH* carrying the plasmid of pSRK-*XccmadB* | This study |
| **Δ***fabH***Δ***madA/*pRP*-EcmadA* | MG1655 *fabH and madA* deletion mutant carrying the plasmid of pRP*-EcmadA* | Laboratory stock |
| **Δ***fabH***Δ***madA/*pRP*-EcmadA* pSRK-*XoomadB* | **Δ***fabH***Δ***madA/*pRP*-EcmadA* carrying the plasmid of pSRK-*XoomadB* | This study |
| **Δ***fabH***Δ***madA/*pRP*-EcmadA* pSRK-*XccmadB* | **Δ***fabH***Δ***madA/*pRP*-EcmadA* carrying the plasmid of pSRK-*XccmadB* | This study |
| ***Ralstonia solanacearum*** | | |
| GMI1000 | Cm^r^, Wild-type | (1) |
| **Δ***RsfabH* | Cm^r^, Δ*fabH* deletion mutant | (2) |
| **Δ***RsfabH/EcmadA* | Cm^r^, Tc^r^,**Δ***RsfabH* carrying the plasmid of pSRK-*EcmadA* | This study |
| **Δ***RsfabH/XoomadB* | Cm^r^, Tc^r^, **Δ***RsfabH* carrying the plasmid of pSRK-*XoomadB* | This study |
| **Δ***RsfabH/XccmadB* | Cm^r^, Tc^r^, **Δ***RsfabH* carrying the plasmid of pSRK-*XccmadB* | This study |
| ***Xanthomonas oryzae* pv. *oryzae*** | | |
| PXO99A | Wild-type | (3) |
| *Xoo***Δ***madB* | *XoomadB* deletion mutant | This study |
| **Δ***fabH1***Δ***madB*/pSRK-*EcmadA* | Tc^r^, **Δ***fabH1***Δ***madB* carry with pSRK-*EcmadA* | This study |
| **Δ***fabH1***Δ***madB*/pSRK-*XoofabH1* | Tc^r^, **Δ***fabH1***Δ***madB* carry with pSRK-*XoofabH1* | This study |
| *Xoo***Δ***madB*::*madB* | Gm^r^, *Xoo***Δ***madB* carry with Tn7-*XoomadB* | This study |
| *Xoo***Δ***rpfBC* | *Xoo* *rpfB rpfC* triple-deletion strain | Laboratory stock |
| *Xoo***Δ***rpfBCmadB* | *Xoo* *rpfB rpfC madB* double-deletion strain | This study |
| *Xoo***Δ***rpfBCmadB*::*madB* | Gm^r^, *Xoo***Δ***rpfBCmadB* carry with Tn7-*XoomadB* | This study |
| *Xoo*/pFA2-P*xoo*316 | Amp^r^, *Xoo* carry with pFA2-P*xoo*316 | This study |
| *Xoo*/pFA2-P*xoo*198 | Amp^r^, *Xoo* carry with pFA2-P*xoo*198 | This study |
| *Xoo*/pFA2-P*xoo*106 | Amp^r^, *Xoo* carry with pFA2-P*xoo*106 | This study |
| *Xoo*/pFA2-P*xoo*HW1m | Amp^r^, *Xoo* carry with pFA2-P*xoo*HW1m | This study |
| *Xoo*/pFA2-P*xoo*HW2m | Amp^r^, *Xoo* carry with pFA2-P*xoo*HW2m | This study |
| *Xoo*/pFA2-P*xoo*HW3m | Amp^r^, *Xoo* carry with pFA2-P*xoo*HW3m | This study |
| ***Xanthomonas campestris* pv. *campestris*** | | |
| *Xcc* 8004 | Rif^r^, Wild-type | (4) |
| *Xcc***Δ***madB* | Rif^r^, *XccmadB* deletion mutant | This study |
| *Xcc***Δ***madB*::*madB* | Rif^r^, Gm^r^, *Xcc***Δ***madB* carry with Tn7-*XccmadB* | This study |
| *Xcc***Δ***fabH/*pSRK-*XccmadB* | Rif^r^, Gm^r^, *Xcc***Δ***madB* carry with pSRK-*XccmadB* | This study |
| *Xcc***Δ***rpfBC* | Rif^r^, *Xcc* *rpfB rpfC* triple-deletion strain | Laboratory stock |
| *Xcc***Δ***rpfBCmadB* | Rif^r^, *Xcc* *rpfB rpfC madB* double-deletion strain | This study |
| *Xcc***Δ***rpfBCmadB*::*madB* | Rif^r^, Gm^r^, *Xcc***Δ***rpfBCmadB* carry with Tn7-*XoomadB* | This study |
| *Xcc*/pFA2-P*xcc*323 | Rif^r^, Amp^r^, *Xoo* carry with pFA2-P*xcc*323 | This study |
| *Xcc*/pFA2-P*xcc*193 | Rif^r^, Amp^r^, *Xoo* carry with pFA2-P*xcc*193 | This study |
| *Xcc*/pFA2-P*xcc*111 | Rif^r^, Amp^r^, *Xoo* carry with pFA2-P*xcc*111 | This study |
| *Xcc*/pFA2-P*xcc*HW3m | Rif^r^, Amp^r^, *Xoo* carry with pFA2-P*xcc*HW3m | This study |
| **Plasmids** |  |  |
| pET-28(b) | Kan^r^, expression plasmid | Novagen |
| pK18mobsacB | Kan^r^, suicide plasmid | (5) |
| pSRK-Tc | Tc^r^, expression plasmid | (6) |
| pSRK-Gm | Gm^r^, expression plasmid | (6) |
| pRP1028 | Spec^R^, expression plasmid | (7) |
| pUC18-mini-*Tn7*T | Gm^r^, expression plasmid | (8) |
| pTNS2 | Amp^r^, *Tn7* transposase expression | (8) |
| pFA2-Gm-Bla-Flag | Amp^r^, β-lactamase expression plasmid | Laboratory stock |
| pRP*-EcmadA* | Spc^r^, *EcmadA* expression plasmid derived from pRP1028 | Laboratory stock |
| pSRK-*EcmadA* | *EcmadA* expression plasmid derived from pSRK-Tc | This study |
| pSRK-*XoomadB* | *XoomadB* expression plasmid derived from pSRK-Tc | This study |
| pSRK-*XccmadB* | *XccmadB* expression plasmid derived from pSRK-Tc | This study |
| pET-*XoomadB* | *XoomadB* expression plasmid derived from pET28(b) | This study |
| pET-*XccmadB* | *XccmadB* expression plasmid derived from pET28(b) | This study |
| Tn7-*XoomadB* | *XoomadB* with *XoomadB*-derived promoter cloned in mini-Tn7T-Gm | This study |
| Tn7-*XccmadB* | *XccmadB* with *XccmadB*-derived promoter cloned in mini-Tn7T-Gm | This study |
| pMF-8 | *XoofabH1* knockout cassette in vector pK18mobsacB | This study |
| pMF-11 | *XoomadB* knockout cassette in vector pK18mobsacB | This study |
| pMF-12 | *XccmadB* knockout cassette in vector pK18mobsacB | This study |
| pFA2-P*xoo*316 | *XoomadB*-300bp promoter cloned in pFA2 | This study |
| pFA2-P*xoo*198 | *XoomadB*-182bp promoter cloned in pFA2 | This study |
| pFA2-P*xoo*106 | *XoomadB*-90bp promoter cloned in pFA2 | This study |
| pFA2-P*xcc*323 | *XccmadB*-300bp promoter cloned in pFA2 | This study |
| pFA2-P*xcc*193 | *XccmadB*-170bp promoter cloned in pFA2 | This study |
| pFA2-P*xcc*111 | *XccmadB*-88bp promoter cloned in pFA2 | This study |
| pFA2-P*xoo*HW1m | *XoomadB*-HW1m promoter cloned in pFA2 | This study |
| pFA2-P*xoo*HW2m | *XoomadB*-HW2m promoter cloned in pFA2 | This study |
| pFA2-P*xoo*HW3m | *XoomadB*-HW3m promoter cloned in pFA2 | This study |
| pFA2-P*xcc*HW3m | *XccmadB*-HW3m promoter cloned in pFA2 | This study |

**Table S2 Sequences of PCR primers used in this study.**

| **Primer name** | **Primer sequence (5′ to 3′)** | **Digestion sites^a^/use** |
| --- | --- | --- |
| *XoofabH1* P1 | AATTGAATTCTCGTTCGCACGATGCCCTGAC | *Eco*R I |
| *XoofabH1* P2 | GAATCAGCCAATCCAGGTCGTTCGACGGAAATATCAGGT |  |
| *XoofabH1* p3 | ACCTGATATTTCCGTCGAACGACCTGGATTGGCTGATTC |  |
| *XoofabH1* p4 | TATAAAGCTTACAAGCAAGGCGACGAAGATG | *Hind* III |
| *XccfabH* P1 | AATTGAATTCGCCAGCGCAGCCTGCAGC | *Eco*R I |
| *XccfabH* P2 | GCAGATCTAGACGGATCCCATATGGTTCCTTGGTGCAAGAGCCG |  |
| *XccfabH* p3 | AACCATATGGGATCCGTCTAGATCTGCGCAGGCACTGCCG |  |
| *XccfabH* p4 | AATTAAGCTTGTAGATTCGGTCACGCGTTG | *Hind* III |
| *XoomadB* P1 | ACTGAATTCAGGCTGGTCTGGCGATAGC | *Eco*R I |
| *XoomadB* P2 | CATGCGGTACCGCATGTCTGACCCTGCAACTGCAATTG |  |
| *XoomadB* p3 | CAATTGCAGTTGCAGGGTCAGACATGCGGTACCGCATG |  |
| *XoomadB* p4 | ATCGGATCCTATTGCGGTTGATCACCCG | *Bam*H I |
| *XccmadB* P1 | ATCGGATCCACCGCATCAAGCTCGAAGGC | *Bam*H I |
| *XccmadB* P2 | GTCTGCCACCAGCTCTTCGTAGACGGCGAAGGGTTGGCATCAT |  |
| *XccmadB* p3 | ATGATGCCAACCCTTCGCCGTCTACGAAGAGCTGGTGGCAGAC |  |
| *XccmadB* p4 | GCAAGCTTTATTGCGGTTGATCACCCGC | *Hin*d III |
| *XoomadB* up1 | ATCGGATCCTGCGGGCTTGCTCCAGAATC | *Bam*H I |
| *XoomadB* up2 | TATATACCATATGACTCCTTCGTGCACGCTCG | *Nde* I |
| *XoomadB* dn | AATTAAGCTTCTACCGCTTTGGGAACGC | *Hin*d III |
| *XccmadB* up1 | AATTATGGGATCCAGTTCTTCCACACGGCGCCCGA | *Bam*H I |
| *XccmadB* up2 | TATATACCATATGACCCTTCCGTGCACGC | *Nde* I |
| *XccmadB* dn | AATTAAGCTTCTACCGTTTTGGAAAGGC | *Hin*d III |
| *XoomadB* up3 | TGCTCTAGACATCGGCAAACACTGAAAGACG | *Xba* I |
| *XoomadB* dn3 | CCCAAGCTTGGCCAGTGCGAGATCTCC | *Hin*d III |
| *XccmadB* up3 | TGCTCTAGACATCGGCAAAGGCTGCAAG | *Xba* I |
| *XccmadB* dn3 | CCCAAGCTTCGCATCAAGCTCGAAGGC | *Hin*d III |
| *XoomadB* 5RACE R3 | GCCGAACGCATTGGCCTTGTCGTTG |  |
| *XoomadB*  5RACE R4 | CGCAGCGCACCATCGTGCAAACC |  |
| *XccmadB* 5RACE R3 | CATCAGCGAGGCCAGACTGCCG |  |
| *XccmadB* 5RACE R4 | GCAGTCGCAAGGCCCCGTCATGC |  |
| RTXomadB P1 | GATCGTGAACAGGTTCTTAG |  |
| RTXomadB P2 | CATCGACAGCACCGGTG |  |
| RTXccmadB P1 | GCAATTGTCGACTTACCAGC |  |
| RTXccmadB P2 | GCAGTCGCAAGGCCCCGTCATGC |  |
| P*xccmadB*-323 P1 | CCCAAGCTTCAATTGTCGACTTACCAGC | *Hin*d III |
| P*xccmadB*-193 P1 | CCCAAGCTTCGCAATGGAAGGCCAAG | *Hin*d III |
| P*xccmadB*-111 P1 | CCCAAGCTTCTGCATGAGAGCAGTTTTAC | *Hin*d III |
| P*xccmadB* P2 | CCGGTCTAGACATCGGCAAAGGCTGCAAGACG | *Xba* I |
| P*xccmadB*HW3m P3 | CTGCATGAGAGCAGTTTTACTCATTTAGGTGCACAAGAC |  |
| P*xccmadB*HW3m P4 | GTCTTGTGCACCTAAATGAGTAAAACTGCTCTCATGCAG |  |
| P*xoomadB*-316 P1 | CCCAAGCTTGGATAGTTCTTCTCGCGC | *Hin*d III |
| P*xoomadB*-198 P1 | CCCAAGCTTGCAACGTTGCGCGCCATC | *Hin*d III |
| P*xoomadB*-106 P1 | CCCAAGCTTCGATGGCTGCATTCAC | *Hin*d III |
| P*xoomadB* P2 | GGTCTAGACATCGGCAAACACTGAAAGACGG | *Xba* I |
| P*xoomadB*HW1m P3 | GTGCTGATGGCGCGCACAGCCATCCGGTCTGGCATGAGG |  |
| P*xoomadB*HW1m P4 | CCTCATGCCAGACCGGATGGCTGTGCGCGCCATCAGCAC |  |
| P*xoomadB*HW2m P3 | GACTTCGAAGCGAAGCCAACCCTGCATTCACCATGCGGTA |  |
| P*xoomadB*HW2m P4 | TACCGCATGGTGAATGCAGGGTTGGCTTCGCTTCGAAGTC |  |
| P*xoomadB*HW3m P3 | TGCGGCGATGGCTGCATTCACAAGTGCACAACACGCCTGCAG |  |
| P*xoomadB*HW3m P4 | CTGCAGGCGTGTTGTGCACTTGTGAATGCAGCCATCGCCGCA |  |

*^a^* Underlined nucleotide sequences are digestion sites of restriction endonucleases.

**Table S3 Annotation of proteins referred in Figure 1 involved in Mad homologue.**

| **Protein** | **Accession number^a^** | **Description** |
| --- | --- | --- |
| *Ec* MadA | b3888 | putative fatty acid biosynthesis enzyme FabY |
| *Pput* MadB | Pput_0277 | thioesterase domain, putative |
| *Xoo* MadB | PXO_03973 | thioesterase domain, putative subfamily |
| *Xcc* MadB | XC_0210 | conserved hypothetical protein |

*^a^* Protein data is sourced from the KEGG database.

**Figures**

**
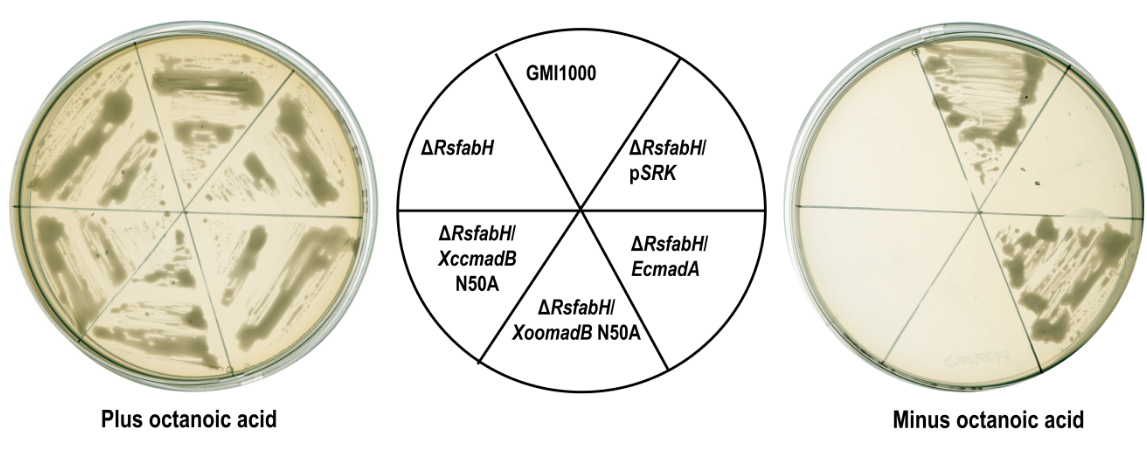
**

**Fig S1** Complementation of *R. solanacearum* Δ*RsfabH* strain with mutated *madB* gene of *Xoo* or *Xcc*. Asn50 (numbered according to the amino acid sequence of *Xoo* or *Xcc* MadB) was substituted with Ala using gene site-directed mutagenesis, and the mutated *madBs* was inserted into pSRK-Tc.

**
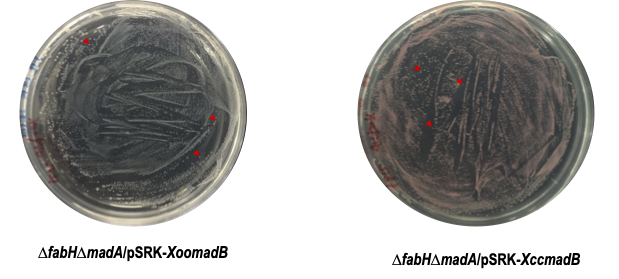
**

**Fig S2** Screening of ∆*fabH*∆*madA* double mutant strains of *E. coli* after expressing *Xoo madB* or *Xcc madB*. The recombinant plasmids pSRK-XoomadB and pSRK-XccmadB were introduced into the *E. coli* ∆*fabH*∆*madA*/pRP-EcmadA strain, and the resulting transformants were spread onto LB plates containing the appropriate antibiotics. The plates were then incubated overnight at 42°C, after which white colonies were screened. The red arrows indicate white colonies.


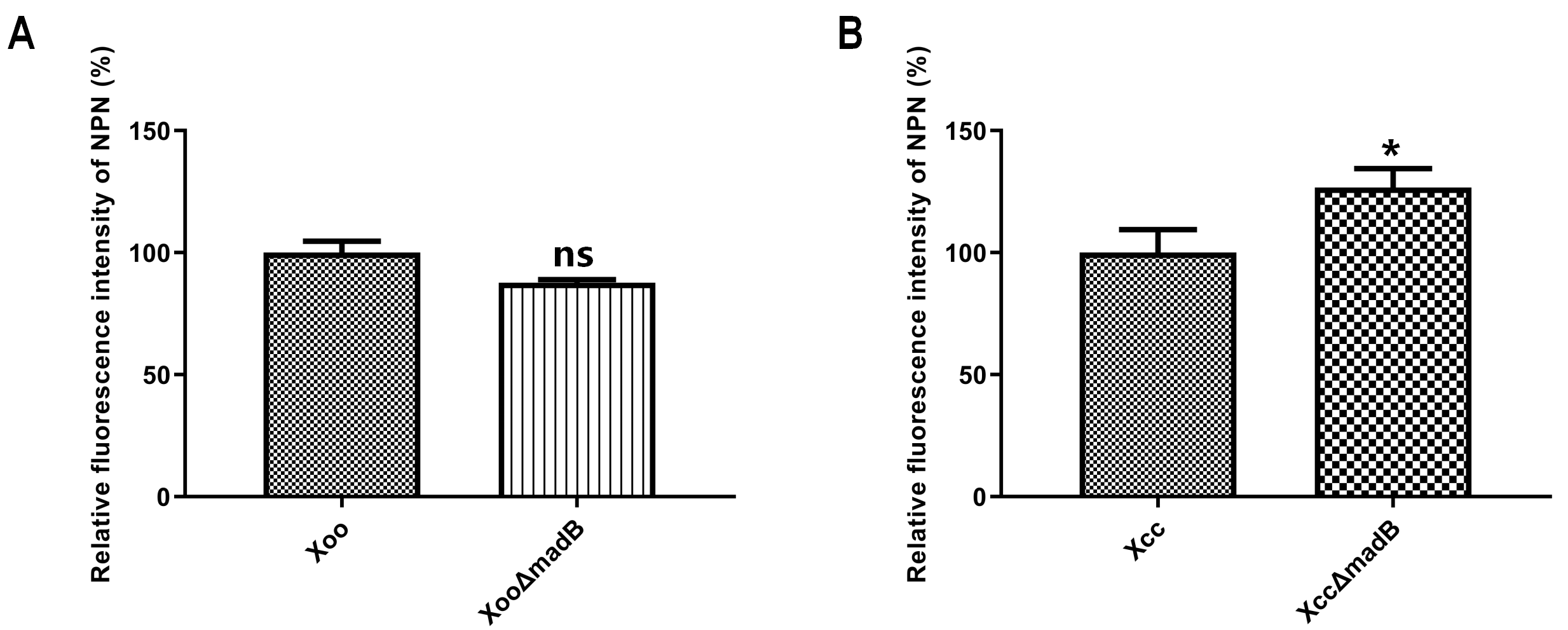
**Fig S3** NPN fluorescence intensity as indicator of the outer membrane permeability of *Xoo* (A) and *Xcc* *madB* (B) mutants*.* The statistical analyses were performed in GraphPad Prism7 with multiple comparisons performed by ordinary one-way ANOVA. The values are presented as the mean ± SD based on the three independent experiments. The asterisks above the error bars indicate significant differences compared with the wild-type strain (*t* test, ^*^*P* < 0.05). All experiments were repeated three times with similar results.

**
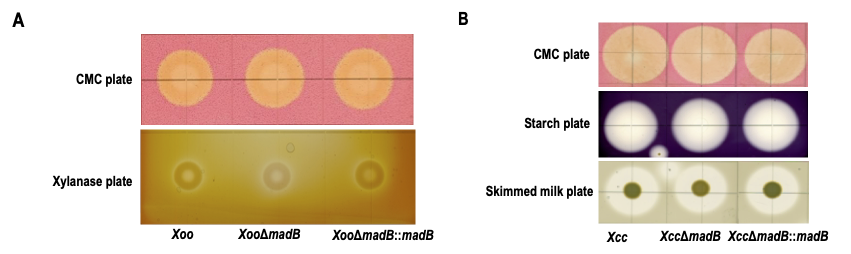
**

**Fig S4** The production of extracellular enzymes by *Xoo* or *Xcc* strains. (A) Extracellular enzymes (cellulase and xylanase) produced by *Xoo* strains. (B) Extracellular enzymes (cellulase, amylase, and protease) produced by *Xcc* strains.

**
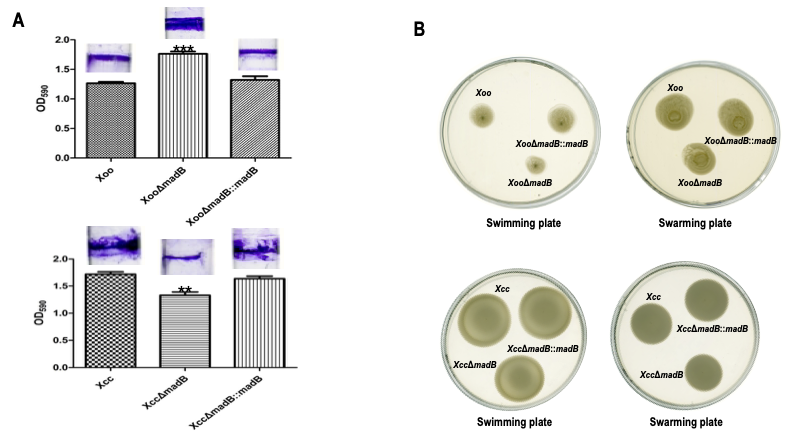
**

**Fig S5** Biofilm formation and cell motility of *madB* mutant strains. (A) Biofilm formation by *Xoo* and *Xcc* *madB* mutants. (B) Patterns of cell motility of *madB* mutant strains on semi-solid agar plates. The statistical analyses were performed in GraphPad Prism7 with multiple comparisons performed by ordinary one-way ANOVA. The values are presented as the mean ± SD based on the three independent experiments. The asterisks above the error bars indicate significant differences compared with the wild-type strain (*t* test, ^**^*P* < 0.01, ^***^*P* < 0.001). All experiments were repeated three times with similar results.

**
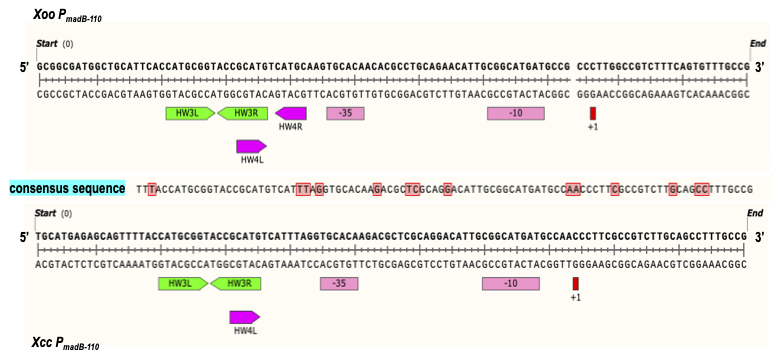
**

**Fig S6** Sequence alignment of the upstream 100 bp DNA regions of *Xoo madB* and *Xcc madB*. HW3, palindromic sequences 3; HW4, palindromic sequences 4.

**
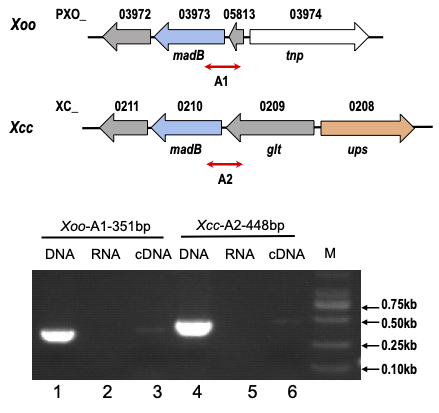
**

**Fig S7** Co-transcription analysis of *madB* and upstream genes with RT-PCR.

**REFERENCES**

1. Salanoubat M, Genin S, Artiguenave F, Gouzy J, Mangenot S, Arlat M, Billault A, Brottier P, Camus JC, Cattolico L, Chandler M, Choisne N, Claudel-Renard C, Cunnac S, Demange N, Gaspin C, Lavie M, Moisan A, Robert C, Saurin W, Schiex T, Siguier P, Thébault P, Whalen M, Wincker P, Levy M, Weissenbach J, Boucher CA. 2002. Genome sequence of the plant pathogen *Ralstonia solanacearum*. Nature 415:497-502. https://doi.org/10.1038/415497a

2. Mao YH, Ma JC, Li F, Hu Z, Wang HH. 2015. *Ralstonia solanacearum* RSp0194 encodes a novel 3-keto-acyl carrier protein synthase III. PloS one 10:e0136261. <https://doi.org/10.1371/journal.pone.0136261>

3. Salzberg SL, Sommer DD, Schatz MC, Phillippy AM, Rabinowicz PD, Tsuge S, Furutani A, Ochiai H, Delcher AL, Kelley D, Madupu R, Puiu D, Radune D, Shumway M, Trapnell C, Aparna G, Jha G, Pandey A, Patil PB, Ishihara H, Meyer DF, Szurek B, Verdier V, Koebnik R, Dow JM, Ryan RP, Hirata H, Tsuyumu S, Won Lee S, Seo YS, Sriariyanum M, Ronald PC, Sonti RV, Van Sluys MA, Leach JE, White FF, Bogdanove AJ. 2008. Genome sequence and rapid evolution of the rice pathogen *Xanthomonas oryzae* pv. *oryzae* PXO99A. BMC Genomics 9:204. https://doi.org/10.1186/1471-2164-9-204

4. Qian W, Jia Y, Ren SX, He YQ, Feng JX, Lu LF, Sun Q, Ying G, Tang DJ, Tang H, Wu W, Hao P, Wang L, Jiang BL, Zeng S, Gu WY, Lu G, Rong L, Tian Y, Yao Z, Fu G, Chen B, Fang R, Qiang B, Chen Z, Zhao GP, Tang JL, He C. 2005. Comparative and functional genomic analyses of the pathogenicity of phytopathogen *Xanthomonas campestris* pv. *campestris*. Genome Res 15(6):757-767. https://doi.org/10.1101/gr.3378705

5. Schäfer A, Tauch A, Jäger W, Kalinowski J, Thierbach G, Pühler A. 1994. Small mobilizable multipurpose cloning vectors derived from the *Escherichia coli* plasmids pK18 and pK19: selection of defined deletions in the chromosome of *Corynebacterium glutamicum*. Gene 145:69–73. https://doi.org/10.1016/0378-1119(94)90324-7

6. Khan SR, Gaines J, Roop RMII, Farrand SK. 2008. Broad-host-range expression vectors with tightly regulated promoters and their use to examine the influence of TraR and TraM expression on Ti plasmid quorum sensing. Appl Environ Microbiol 74:5053–5062. https://doi.org/10.1128/AEM.01098-08

7. Su S, Bangar H, Saldanha R, Pemberton A, Aronow B, Dean GE, Lamkin TJ, Hassett DJ. 2014. Construction and characterization of stable, constitutively expressed, chromosomal green and red fluorescent transcriptional fusions in the select agents, *Bacillus anthracis*, *Yersinia pestis*, *Burkholderia mallei*, and *Burkholderia pseudomallei*. Microbiologyopen 3(5):610-629. https://doi.org/10.1002/mbo3.192

8. Choi KH, Gaynor JB, White KG, Lopez C, Bosio CM, Karkhoff-Schweizer RR, Schweizer HP. 2005. A Tn*7*-based broad-range bacterial cloning and expression system. Nat Methods 2(6):443-448. https://doi.org/10.1038/nmeth765
